# Inhibition of interferon-gamma-stimulated melanoma progression by targeting neuronal nitric oxide synthase (nNOS)

**DOI:** 10.1038/s41598-022-05394-6

**Published:** 2022-02-01

**Authors:** Shirley Tong, Maris A. Cinelli, Naglaa Salem El-Sayed, He Huang, Anika Patel, Richard B. Silverman, Sun Yang

**Affiliations:** 1 Department of Pharmacy Practice, Chapman University School of Pharmacy, Harry and Diane Rinker Health Science Campus, #297-Y, 9401 Jeronimo Road, Irvine, CA 92618 USA; 2 Department of Chemistry, Department of Molecular Biosciences, Chemistry of Life Processes Institute, Center for Molecular Innovation and Drug Discovery, and Center for Developmental Therapeutics, Northwestern University, Evanston, IL 60208 USA; 3 Department of Pharmacology, Feinberg School of Medicine, Northwestern University, Chicago, IL 60611 USA

**Keywords:** Melanoma, Drug discovery, Pharmacology, Immunosuppression

## Abstract

Interferon-gamma (IFN-γ) is shown to stimulate melanoma development and progression. However, the underlying mechanism has not been completely defined. Our study aimed to determine the role of neuronal nitric oxide synthase (nNOS)-mediated signaling in IFN-γ-stimulated melanoma progression and the anti-melanoma effects of novel nNOS inhibitors. Our study shows that IFN-γ markedly induced the expression levels of nNOS in melanoma cells associated with increased intracellular nitric oxide (NO) levels. Co-treatment with novel nNOS inhibitors effectively alleviated IFN-γ-activated STAT1/3. Further, reverse phase protein array (RPPA) analysis demonstrated that IFN-γ induced the expression of HIF1α, c-Myc, and programmed death-ligand 1 (PD-L1), in contrast to IFN-α. Blocking the nNOS-mediated signaling pathway using nNOS-selective inhibitors was shown to effectively diminish IFN-γ-induced PD-L1 expression in melanoma cells. Using a human melanoma xenograft mouse model, the in vivo studies revealed that IFN-γ increased tumor growth compared to control, which was inhibited by the co-administration of nNOS inhibitor MAC-3-190. Another nNOS inhibitor, HH044, was shown to effectively inhibit in vivo tumor growth and was associated with reduced PD-L1 expression levels in melanoma xenografts. Our study demonstrates the important role of nNOS-mediated NO signaling in IFN-γ-stimulated melanoma progression. Targeting nNOS using highly selective small molecular inhibitors is a unique and effective strategy to improve melanoma treatment.

## Introduction

Human cutaneous melanoma (CM) incidence rates continue to increase in recent decades, making this disease a rising public health concern. With a high rate of genomic mutations^1^ and diverse resistance mechanisms exploited by the disease, melanoma remains the deadliest and most aggressive form of skin cancer^2^. Despite the exciting developments in melanoma therapy, the clinical responses to newly developed targeted therapy using small molecular inhibitors is significantly limited due to unavoidable acquired resistance^3^. A large population of melanoma patients failed to respond or discontinued the current revolutionary immunotherapy due to severe adverse events^4^. As such, the development of novel therapeutic interventions to block melanomagenesis and disease progression to advanced stages has both high impact and significance.

It has been well-documented that UVR, a major environmental contributing factor involved in melanomagenesis and disease progression, causes human skin to exhibit a remarkable increase in nitric oxide (NO)^5^. In recent years, more studies have revealed the role of NO in tumor development and progression including melanoma^6,7^. The nitric oxide synthase (NOS) enzymes produce NO from L-arginine and are composed of inducible NOS (iNOS), endothelial NOS (eNOS), and neuronal NOS (nNOS). Melanocytes originate from the neural crest and have many gene expression characteristics similar to neural cells^8^. Since nNOS is expressed primarily in neuronal tissue, nNOS plays a prominent role in regulating NO levels in melanocytes^9^. Our previous studies on patient biopsies have also shown that compared to normal skin, malignant melanomas exhibited markedly higher expression levels of nNOS, which is significantly correlated with the disease stage^10^. In a recent study reported by Liu et al. (2014), elevated nNOS expression in human melanoma tissue was linked to immune dysfunction of circulating T lymphocytes, resulting in immunosuppression^11^. Although further mechanistic studies are warranted, this explorative observational study revealed an important role of nNOS-mediated NO signaling in regulating immune response, particularly for human melanoma.

Interferon-gamma (IFN-γ), a key immunoregulatory cytokine, exhibits profound stimulation of T cell immunity, which is commonly involved in the reduction of the frequency and severity of serious infections associated with chronic granulomatous disease. However, preclinical studies in cancer demonstrated an enhancement of tumor metastasis potential with IFN-γ exposure, which is consistent in several different model systems as described previously, including melanoma^12,13^. A study in the UVB-HGF/SF transgenic mouse melanoma model^14^, demonstrated the direct involvement of macrophage-generated IFN-γ in promoting melanoma growth by inhibiting apoptosis^15^. Specific antibodies blocking IFN-γ, but not IFN-α, abolished the UVB-induced melanocyte activation. It is also proposed that depending on the context of micro-environmental factors, the role of IFN-γ may switch from immune surveillance to immune editing^16^. In fact, an earlier Southwest Oncology Group (SWOG) clinical trial done in 1990, showed that IFN-γ treatment stimulated disease progression in early-stage melanoma leading to more than 50% of patients relapsing or expiring. Although a study in B16 mouse melanoma cells suggested a beneficial effect of IFN-γ in inhibiting metastasis and reducing tumor development^17^, the relevance of this mouse model to the human disease is, however, quite weak. There is emerging evidence indicating potential interactions between IFN-γ signaling and PD-L1-mediated immunosuppression in melanoma^18^. Further mechanistic studies identified the binding sites of STAT1/3, which are downstream of IFN-γ signaling, present on the PD-L1 promoter^19^. In another study, IFN-γ was shown to induce the expression of PD-L1 in human melanoma cells in a NF-kB-dependent manner^18^.

To date, the underlying molecular mechanisms of IFN-γ-mediated pro-tumorigenesis have not been well defined. We hypothesize that IFN-γ may alter the immune microenvironment of melanoma cells either directly by the nNOS/NO pathway or indirectly by potentializing PD-L1-mediated immune inhibition. Our study focuses on demonstrating the underlying mechanisms of IFN-γ-stimulated melanoma progression and developing novel inhibitors targeting the IFN-γ-mediated signaling pathway for melanoma prevention and therapy. Our studies will not only enhance the fundamental understanding of melanoma pathogenesis but may also lead to the development of treatments that complement existing immunotherapy with immune checkpoint inhibitors.

## Results

### IFN-γ stimulates melanoma progression via activation of nNOS-NO signaling

**Figure 1:**
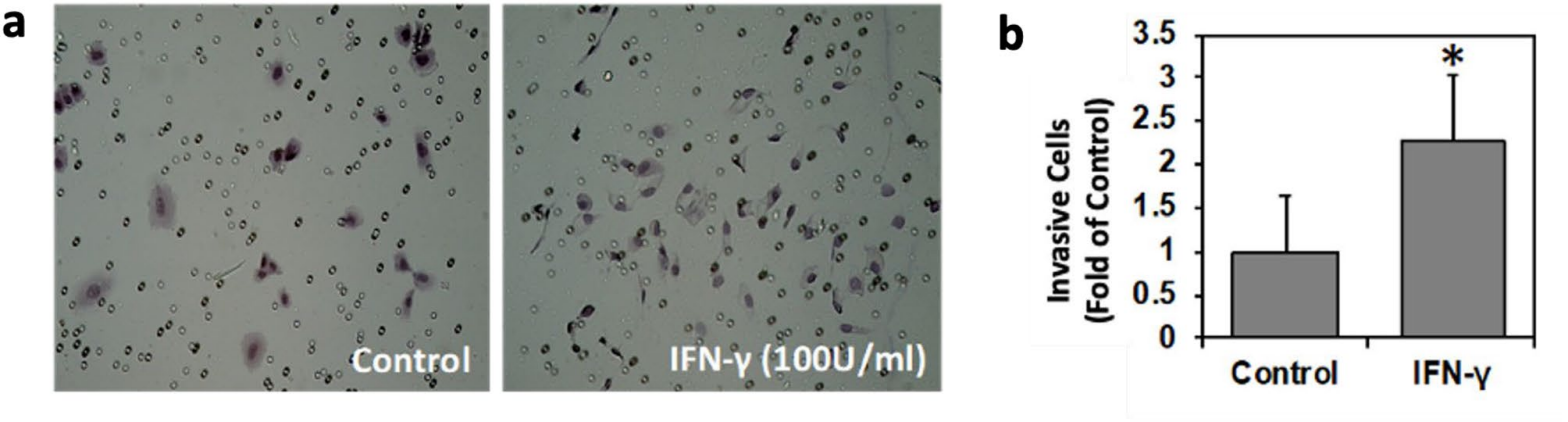
IFN-γ increased melanoma invasion potential as detected by matrigel invasion assay. The represented image (**a**) was from A375 metastatic melanoma cells treated with IFN-γ (100 units/mL) for 16 h. The invasive cells that traversed the Matrigel and spread to the lower surface of the polyethylene filter membrane were stained with hematoxylin and eosin. The numbers in 10 vision fields were counted using a light microscope. (**b**) The invasive cell numbers after IFN-γ treatment increased compared to control. *p < 0.05 compared to control.

**Figure 2:**
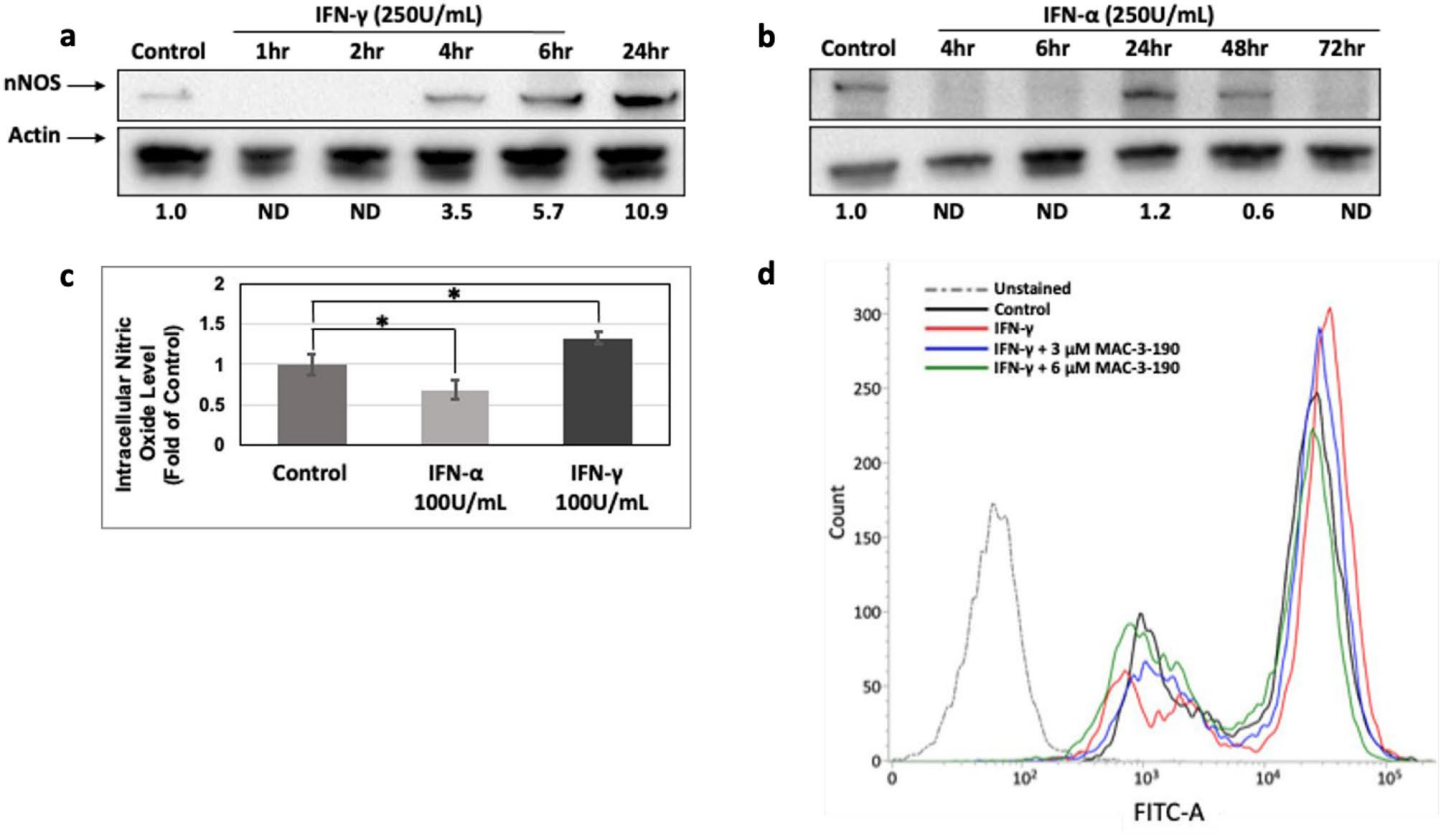
(**a,b**) Effects of IFN-α and IFN-γ treatment on nNOS expression levels in human metastatic melanoma A375 cells. Cells were treated with IFN-α or IFN-γ for various timepoints and whole cell lysates were collected. Samples were subjected to Western blot analysis for nNOS. A control of the protein loading was performed by detecting actin. Full length blots are presented in Supplemental Figure S2a,b, respectively. (**c)** Intracellular nitric oxide levels of A375 cells detected with a microplate reader using a DAF-FM fluorescence probe after IFN-α or IFN-γ (100 units/mL) treatment for 24 h, respectively. *p < 0.05 compared to control. (**d)** nNOS inhibitor reduced intracellular nitric oxide levels in A375 cells. Cells treated with 250 units/mL of IFN-γ with or without nNOS inhibitor, MAC-3-190, for 4 h followed by flow cytometry analysis.

Utilizing metastatic human melanoma A375 cells, we determined the effect of IFN-γ on melanoma invasion potential. As shown in Fig. 1, IFN-γ significantly enhanced melanoma invasion potential compared to control (*p* < 0.05). IFN-γ markedly induced nNOS expression levels in metastatic melanoma A375 cells (Fig. 2a). The induction of nNOS occurred as early as 4 h and persists for more than 24 h after exposure. In contrast, the expression of nNOS rapidly dropped to undetectable levels after IFN-α treatment within 4 h, which was then recovered by 24 h (Fig. 2b). In parallel with increased nNOS expression, elevated intracellular NO levels in A375 cells were also detected using DAF-FM fluorescence probe after exposure to IFN-γ (100 units/mL) for 24 h. However, in IFN-α treated cells, NO production was significantly decreased compared to that of control (Fig. 2c), which is consistent with the reduced nNOS expression after IFN-α treatment (Fig. 2b). In addition, cotreatment of A375 cells with nNOS inhibitor, MAC-3-190, effectively inhibited intracellular NO levels induced by IFN-γ (Fig. 2d).

**Figure 3:**
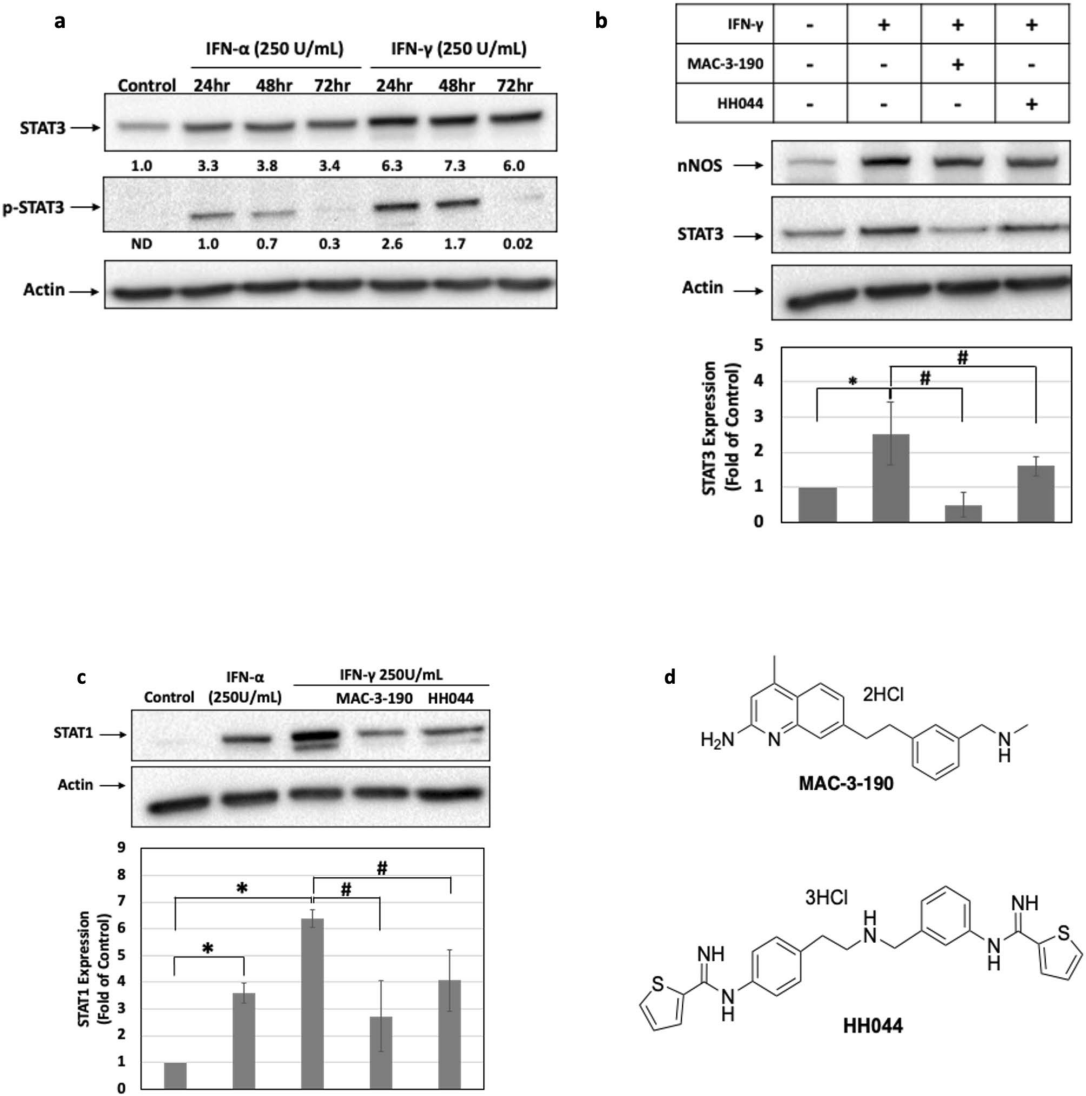
(**a**) Effects of IFN-γ on STAT3 and phospho-STAT3 expression levels in melanoma. Primary melanoma wm115 cells were treated with IFN-γ or IFN-α (250 units/mL) for various timepoints. Whole cell lysates were collected for Western blot analysis to detect STAT3 and p-STAT3 levels, respectively. Specific nNOS inhibitor MAC-3-190 (3 µM) inhibited the activation of STAT3 **(b)** and STAT1 **(c)** expressions induced by IFN-γ. A375 cells were treated with IFN-γ (250 units/mL) with or without MAC-3-190 for 48 h. Shown is the mean ± SD, n = 3 for each experiment, *p < 0.05 compared to control, and #*p* < 0.05 compared to IFN-γ treatment. Full length blots are presented in Supplementary Figure S3a-c, respectively. (**d**) Chemical structures of novel nNOS inhibitors MAC-3-190 and HH044.

As a downstream target of the IFN-γ signaling pathway, the expression levels of STAT3 were increased to 6.3-fold of control when wm115 primary melanoma cells were exposed to IFN-γ (250 units/mL) for 24 h. Phospho-STAT3 levels were also markedly elevated, suggesting that IFN-γ treatment was associated with activation of STAT3-mediated signaling (Fig. 3a). Such induction was also observed at lower concentrations of IFN-γ (50 and 100 units/mL; Fig. S3d,e). The inductions of STAT3 and phospho-STAT3 were also observed after IFN-α treatment but to a lesser degree. Of note, nNOS inhibitors, MAC-3-190 and HH044, failed to inhibit IFN-γ induced nNOS expression. However, the co-treatment of MAC-3-190 (3 µM) effectively diminished the induction of STAT3 expression after IFN-γ exposure (Fig. 3b). Though IFN-α was observed to increase the expression levels of STAT1, IFN-γ exhibited a higher potency to induce STAT1 expression at the same concentration (Fig. 3c). The co-treatment with nNOS inhibitors also effectively inhibited the induction of STAT1 by IFN-γ (*p* < 0.05). Structures for nNOS inhibitors, MAC-3-190 and HH044 are as shown in Fig. 3d.

### The induction of PD-L1 expression by IFN-γ was diminished by nNOS inhibitor treatment

**Figure 4:**
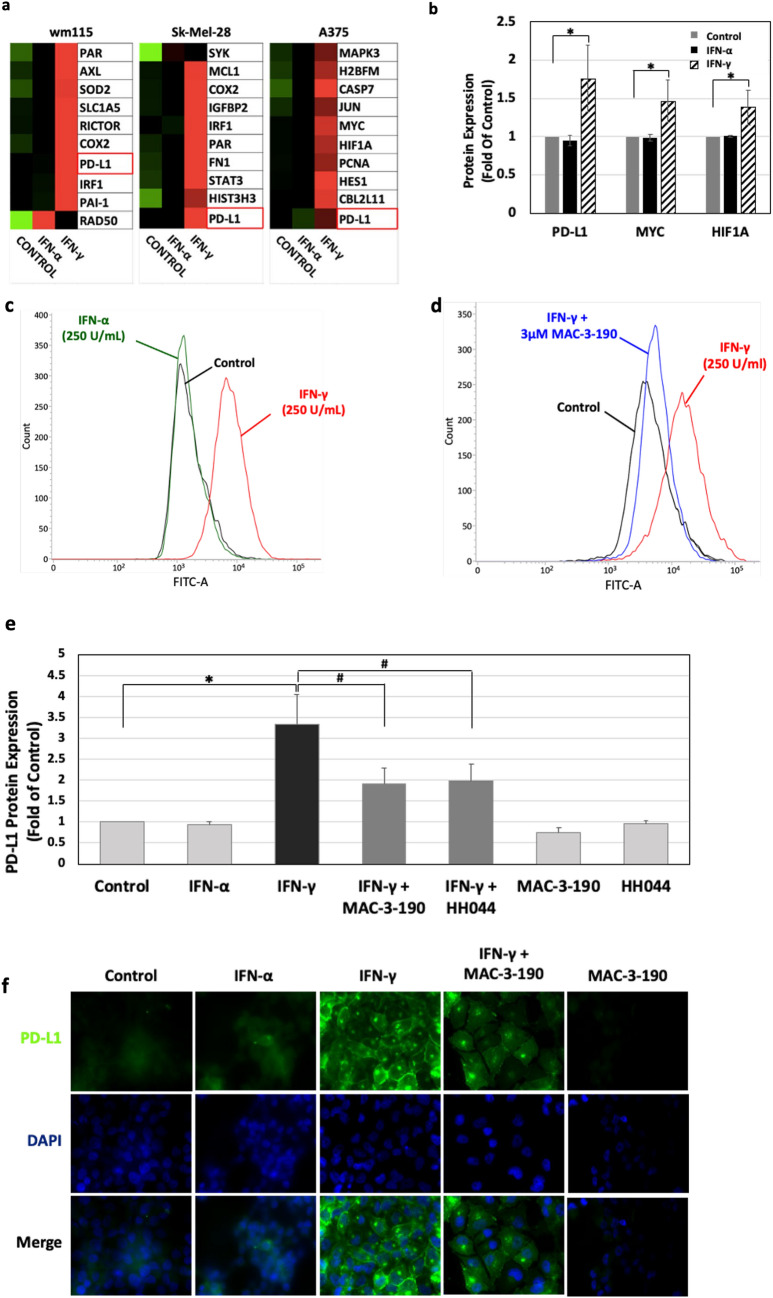
(**a**) Heat map of reverse phase protein array (RPPA) showing distinct effects of IFN-α and IFN-γ on protein expression levels in human melanoma cells. Three melanoma cell lines (wm115, Sk-Mel-28 and A375) were treated with 250 units/mL of interferons for 48 h. Whole cell lysates were collected and subjected to RPPA assay. The top 10 upregulated proteins by IFN-γ were selected from 302 proteins and phosphorylation of key signaling molecules. Red, above median; green, below median. All the data points were normalized for protein loading and transformed to linear values. The heatmaps included in supplemental data were generated in Cluster 3.0 (http://bonsai.hgc.jp/~mdehoon/software/cluster/software.htm) as a hierarchical cluster using Pearson Correlation and a center metric. (**b**) PD-L1, c-Myc and HIF1α were significantly induced by IFN-γ. Average changes of three cell lines detected by RPPA are shown in the figure. *p < 0.05 in comparison to that of control. (**c**) Distinct effects of IFN-α and IFN-γ on the expression levels of PD-L1. Human melanoma A375 cells were incubated with 250 units/mL of IFN-α or IFN-γ for 48 h, followed by detection of PD-L1 levels using flow cytometry. (**d**,**e**) The induction of PD-L1 by IFN-γ was diminished by the co-treatment of nNOS inhibitors. **A375** melanoma cells were exposed to IFN-γ (250 units/mL) with or without 3 µM of nNOS inhibitor MAC-3-190 or HH044 for 48 h. The relative expression of PD-L1 on the cell surface was determined by flow cytometry. Representative histograms out of two independent experimental replicates are shown. *p < 0.05 compared to control; #*p* < 0.05 compared to IFN-γ alone. (**f**) Expression of PD-L1 in metastatic melanoma A375 cells detected by immunofluorescence staining. A375 cells were plated on coverslips and allowed to adhere overnight to 75% confluence then treated with IFN-α or IFN-γ (250 units/mL) with or without MAC-3-190 (3 μM) of 72 h. Cells were then fixed and permeabilized with 4% formaldehyde and methanol. Samples were blocked in blocking buffer containing 5% horse serum for 1 h. The slides were then allowed to incubate in a 1:50 PD-L1 antibody dilution overnight at 4 ºC and DAPI reagent for 1 h. Representative images are shown stained with PD-L1 antibody (green) and DAPI (blue fluorescence) (100× magnification). Representative images for two experimental replicates are shown.

RPPA was used to assess the effects of IFN-α and IFN-γ on the major growth and survival signaling molecules in three human melanoma cell lines (Fig. 4a). Consistent with a recent study^18^, IFN-γ treatment significantly upregulated PD-L1 expression compared to control. Increased c-Myc and HIF-1α expression levels were also evident after IFN-γ treatment (Fig. 4b), which are associated with tumor metastasis and poor prognosis in melanoma patients^20,21^. However, such changes were not observed in melanoma cells after IFN-α exposure (Fig. 4b).

Upregulation of PD-L1 by IFN-γ was further confirmed using flow cytometry. Our data showed that 250 units/mL IFN-γ treatment significantly increased the expression of PD-L1 in all three melanoma cell lines examined, while at the same concentration, IFN-α only exhibited minimal effect on PD-L1 levels (Fig. 4c). Co-treatment with 3 µM nNOS inhibitor MAC-3-190 significantly inhibited the induction of PD-L1 by IFN-γ (Fig. 4d). The relative PD-L1 expression levels after 250 units/mL IFN-γ treatment were decreased from 3.3-fold of control to 1.9-fold and 2.0-fold of control when co-incubated with MAC-3-190 or HH044, respectively (*p < 0.05 compared to control; #*p* < 0.05 compared to IFN-γ alone, Fig. 4e).

As show in Fig. 4f, images obtained from immunofluorescence microscopy, control and IFN-α treated cells showed low basal expression of PD-L1. After incubation with IFN-γ, however, the extracellular expression of PD-L1 was markedly induced in melanoma cells as indicated by the intense green fluorescence staining. Co-treatment with MAC-3-190 (3 μM) significantly reduced the IFN-γ-inducible PD-L1 expression, while treatment with MAC-3-190 alone did not significantly alter the basal level of PD-L1 compared to that of control (Fig. 4f).

### Anti-melanoma activities of nNOS inhibitors in vitro and in a human melanoma xenograft mouse model

**Figure 5:**
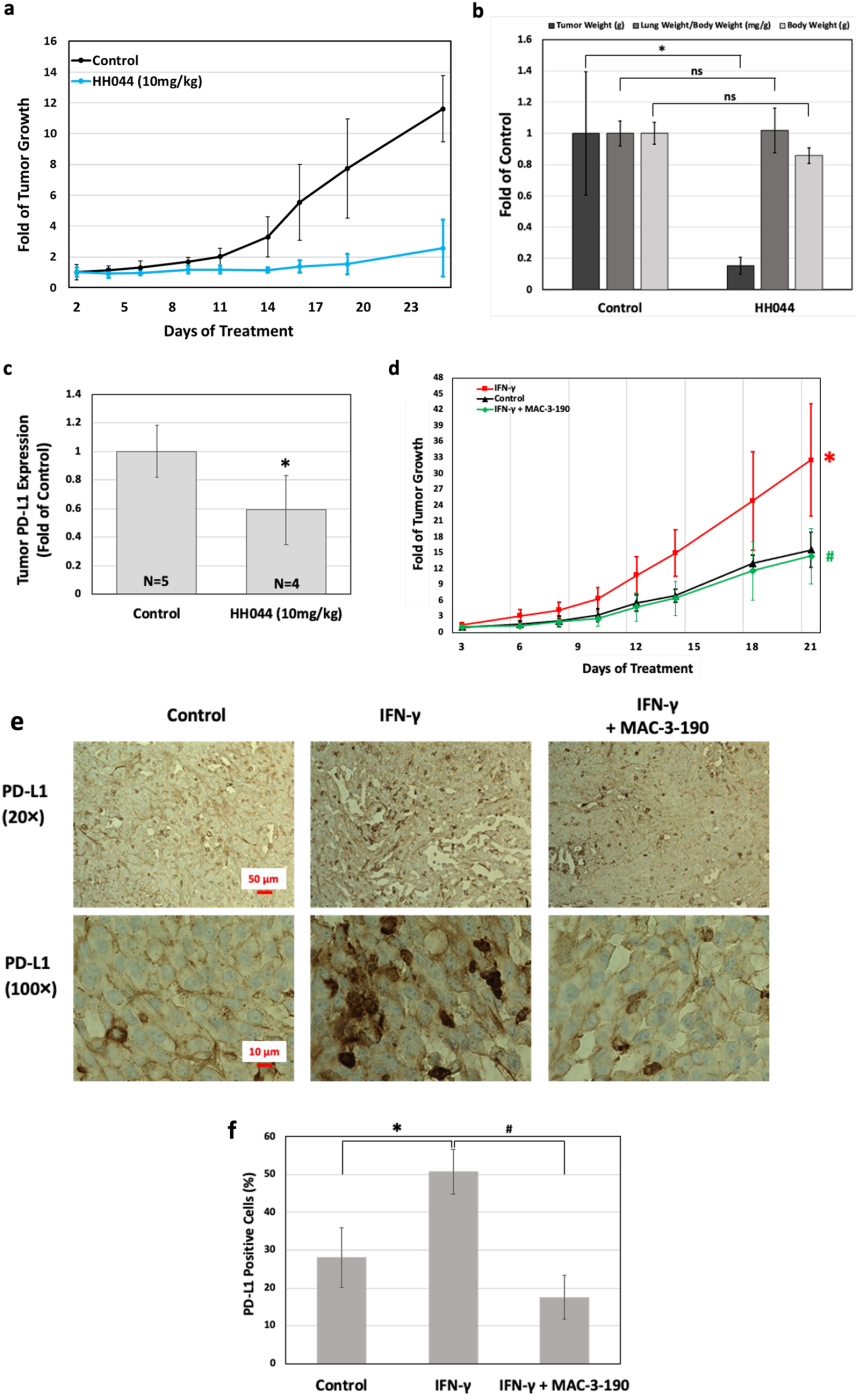
Anti-melanoma activity of novel nNOS inhibitors. Metastatic melanoma A375 cells were injected to nude mice subcutaneously on the flank. The growth of tumor was measured daily and tumor volumes were determined using digital calipers (Fisher Sci) by using the formula tumor volume (mm^3^) = [Length × (Width^2^)]/2. Data are represented as mean ± SD. (**a)** nNOS inhibitor HH044 (10 mg/kg, *i.p.* daily) markedly inhibited the tumor growth of human melanoma in vivo compared to control (Control, n = 5; HH044, n = 4). (**b)** HH044 significantly decreased the final mass of xenograft tumors with no significant change in lung and body weight. *p < 0.05 compared to control; ns, *p* > 0.05, compared to control (Control, n = 5; HH044, n = 4). (**c)** PD-L1 expression of HH044 treated tumors was significantly decreased as detected by flow cytometry. *p < 0.05 compared to control (Control, n = 5; HH044, n = 4). Single cell suspensions of harvested tumor xenografts were stained with Alexa Fluor 488 conjugated PD-L1 antibody. The relative expression levels of PD-L1 were determined by the average fluorescence density as detected by flow cytometry. (**d)** nNOS inhibitor MAC-3-190 (5 mg/kg, *i.p.* daily) diminished the tumor growth stimulated by IFN-γ (1000 units*, i.p.* daily). *p < 0.05 compared to control; #*p* < 0.05 compared to IFN-γ treatment **(**Control, n = 7; IFN-γ, n = 11; IFN-γ + MAC-3-190, n = 5). (**e**,**f**) Expression levels of PD-L1 induced by IFN-γ treatment were inhibited by nNOS inhibitor MAC-3-190 in vivo. Metastatic melanoma A375 cells were injected to nude mice subcutaneously on the flank. IFN-γ (1000 units/day) was injected intraperitoneally once daily and nNOS inhibitor MAC-3-190 was administered *i.p.* daily at a dosing of 5 mg/kg for 21 days. The expression of PD-L1 in xenograft tumor samples were detected by immunohistochemistry staining in T-cell non-infiltrated area. By the end of study, xenograft tumors from different treatment groups were collected and specimens were fixed in a 10% formalin solution and embedded in paraffin wax for automatic processing using the Ventana Benchmark Ultra machine. Images of PD-L1 staining (brown) were captured in CD8-negative areas at 20× and 100× magnification, respectively. PD-L1 positive cells were quantified using ImageJ (https://imagej.nih.gov/ij/index.html) and represented as percentage of PD-L1 positive staining in the graph. Representative sections of each condition are shown. *p < 0.05 compared to control group; #*p* < 0.05, compared to IFN-γ group (n = 4 of each treatment).

The newly developed nNOS inhibitors (HH044 and MAC-3-190) exhibited potent anti-melanoma activity both in vitro (Table 1) and in vivo (Fig. 5). As listed in Table 1, the IC_50_ values of all candidate compounds are less than 10 µM, which are comparable or even more potent in comparison to that of the chemotherapeutic drug cisplatin (4.2 µM and 14.3 µM in A375 and Sk-Mel-28 cells, respectively, unpublished data). Notably, the cytotoxicities of HH044 and MAC-3-190, were not selective to human melanoma cells when compared to immortal melanocytes (Hermes 1, Hermes 3a, and Hermes 4a; Table 1), which also express nNOS as detected by Western Blot (Fig. S1).

**Table 1:** Novel potent and highly selective nNOS inhibitors.

Compounds	Ki (µM)			Selectivity^a^		Cytotoxicity (IC_50_ from 3 melanoma cell lines^b^) (µM)	Cytotoxicity (IC_50_ from 3 immortal melanocyte cell lines^c^)
	nNOS	iNOS	eNOS	nNOS/iNOS	nNOS/eNOS		
HH044^22^	0.005	1.7	2.7	340	540	5.27 ± 3.3	4.25 ± 1.67^NS^
MAC-3-190^23^	0.033	4.5	3.9	138	119	1.21 ± 0.19	1.95 ± 0.87^NS^

All the NOS isozymes used were recombinant enzymes overexpressed in *E. coli*. Ki values are calculated directly using known literature methods and detailed in previously published manuscripts^22–26^. Cytotoxic effects of nNOS inhibitors in human melanoma were detected by MTT colorimetric analysis and compared to that of human immortal melanocytes^24,27^. The IC_50_ values are the average of three cell lines.

^a^Selectivity of nNOS over iNOS or eNOS was calculated as described previously.

^b^Three human melanoma cell lines: A375—metastatic, BRAF^V600E^; Sk-Mel-28—metastatic, BRAF^V600E^; wm3211—primary, BRAF^wt^.

^c^Three human immortal melanocyte cell lines: Hermes 1, Hermes 3a and Hermes 4a.

^NS^No statistical difference compared to IC50s of melanoma cells lines.

Using a human melanoma xenograft tumor model, we further determined the in vivo effects of nNOS inhibitor, HH044, on tumor growth. Treatments with HH044 (10 mg/kg/day *i.p.* for 21 days) significantly reduced tumor growth with no apparent systemic toxicities observed (Fig. 5a,b). As shown in Fig. 5b, it was also found at the end of the study that the mass of the tumor treated with HH044 was significantly reduced (*p* < 0.05 compared to control) with no significant changes in lung and body weight (*p* > 0.05 compared to control). Analysis of single cell suspensions obtained from xenografted tumors showed a significant decrease of PD-L1 expression levels in HH044-treated mice in comparison to the control group (Fig. 5c, *p* < 0.05).

Our animal study also demonstrated that IFN-γ treatment (1000 units/day, *i.p.*) significantly stimulated tumor growth in vivo (Fig. 5d). Co-treatment with MAC-3-190, a water soluble and potent nNOS inhibitor, effectively diminished the induction in tumor volume by the end of study (*p* < 0.05 compared to IFN-γ treatment). This induction of tumor growth by IFN-γ appears to be effectively blocked by the co-administration of MAC-3-190 even at very low dosage (5 mg/kg/day*, i.p.*).

### PD-L1 expression elevated in CD8-negative melanoma tumor when treated with IFN-γ

As shown in the representative images in Fig. 5e,f, the average positive staining of PD-L1 in control A375 xenograft tumors was 28%, which was elevated to 50.6% after 21-day treatment of IFN-γ (1000 units per mouse, *i.p.* daily). Co-treatment with MAC-3-190 (5 mg/kg/day, *i.p*.) effectively decreased PD-L1-positive staining to 17.5% (*p* < 0.05 compared to IFN-γ treatment) (Fig. 5f). Images used to determine average PD-L1 staining can be found in Supplementary Fig. S4. Although the percent PD-L1-positive staining in the co-treatment was lower than in the control group, there is no statistical significance observed (*p* > 0.05). Of note, PD-L1 positive staining was evident in CD8-negative tumor tissues after IFN-γ treatment, which suggests that the induction of PD-L1 was independent of the presence of tumor infiltrated lymphocytes (TILs) and might be stimulated by IFN-γ directly.

## Discussion

**Figure 6:**
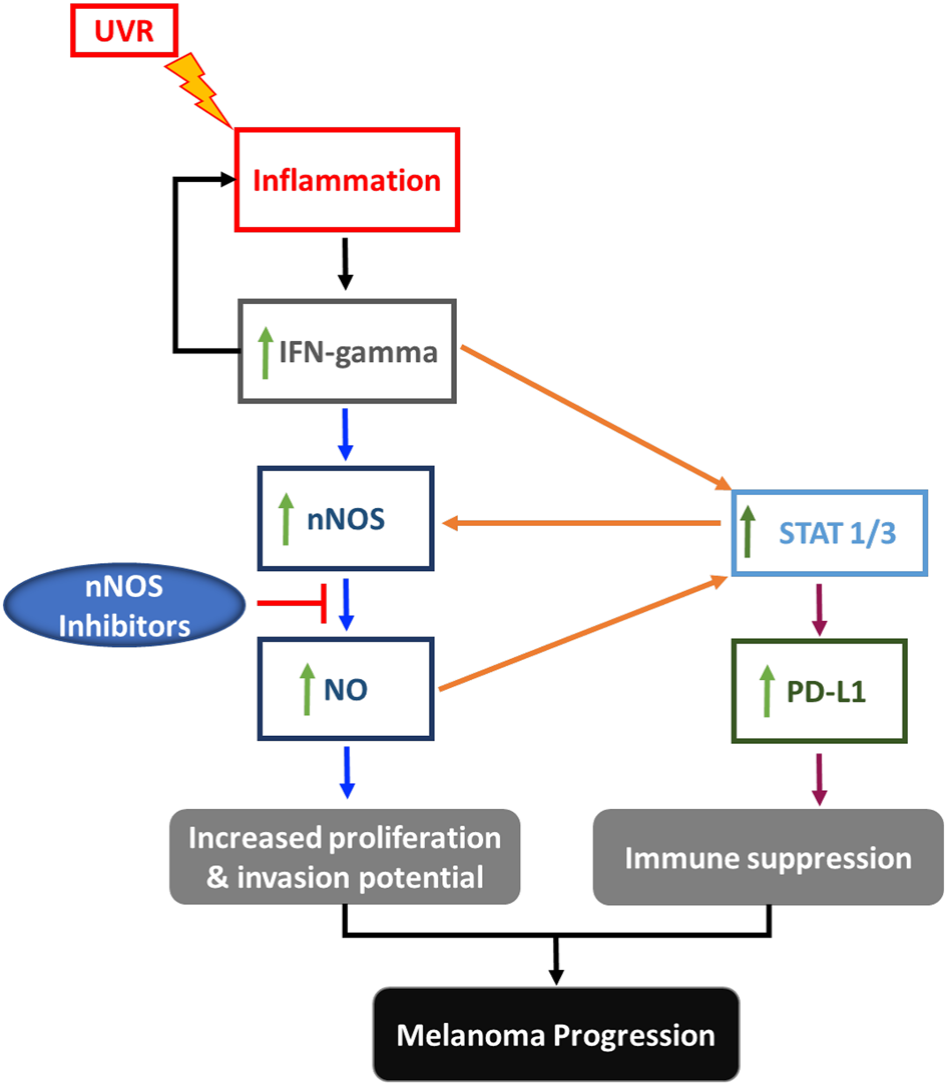
nNOS plays a central role in interferon-γ-mediated melanoma progression. Studies have shown that UV radiation, especially at sunburn doses, causes immunological and inflammatory effects^28^, damages the skin and stimulates the production of IFN-γ^15^. IFN-γ is shown to promote inflammation, melanomagenesis and disease progression both in a transgenic mouse model^15^ and melanoma patients^29^. Our study shows that IFN-γ triggers the activation of nNOS-NO signaling cascades associated with the activation of nuclear transcription factor STAT3. Abnormally high levels of NO fuel melanoma proliferation and facilitate the escape of cancer cells from immune surveillance by inducing the expression of PD-L1, which negatively regulates T-cell responses to tumor cells. nNOS inhibitors not only effectively reduce the production of NO, but also inhibit IFN-γ-stimulated PD-L1 expression and the activation of STAT1/3 signaling. Both in vitro and in vivo studies demonstrate that targeting nNOS-NO using small molecular inhibitors is a promising strategy for melanoma therapy.

Our study demonstrates the critical role of nNOS-mediated NO signaling in IFN-γ-stimulated melanoma progression both in vitro and in vivo. Pro-tumorigenic IFN-γ treatment significantly increased nNOS expression levels in melanoma cells associated with increased intracellular nitric oxide production. nNOS-selective small molecular inhibitors effectively inhibited the induction of PD-L1 stimulated by IFN-γ treatment. Our data also demonstrated that co-treatment with nNOS inhibitors has effectively alleviated the activation of STAT3-signaling after IFN-γ exposure. Consistently, in vivo studies showed that co-treatment with nNOS inhibitor MAC-3-190 effectively suppressed melanoma tumor growth stimulated by IFN-γ in a human melanoma xenograft mouse model. In addition, nNOS inhibitor HH044 significantly inhibited tumor growth and PD-L1 expression in tumor xenografts, indicating a potential role of nNOS-mediated NO signaling in regulating tumor immune responses in vivo. Our study, in combination with accumulating evidence, indicates that targeting nNOS-mediated NO signaling using small molecule inhibitors may be a novel and effective strategy for melanoma therapy (Fig. 6).

Lollini’s group showed that IFN-γ treatment generated a significant increase in tumor metastasis independent of its anti-proliferative effect in mouse melanoma with an approximately 20-fold increase in the number of lung metastases^30^. Our study also observed that IFN-γ significantly increased the invasion and metastatic potential of melanoma cells in vitro (Fig. 1). Consistently, another recent study showed that IFN-γ enhances the expression of CD74, which interacts with its ligand and thereby activates the PI3K/AKT pathway in melanoma, leading to the promotion of tumor survival and growth^31^. This remarkable pro-tumorigenic activity of IFN-γ in human melanoma was also observed in an earlier Phase III clinical trial, which showed that adjuvant treatment with daily subcutaneous injection of IFN-γ failed to improve disease-free survival or overall survival of patients with high-risk melanoma resected with curative intent, constituting strong evidence against any clinically beneficial application^32^. The molecular mechanisms of IFN-γ-mediated pro-tumorigenic effects, however, are not yet fully understood. The distinct responses of IFN-γ in melanoma compared to other tumors suggest that IFN-γ might activate a unique signaling pathway, facilitating the progression of disease.

Distinct from IFN-γ, which is pro-tumorigenic and stimulates melanoma progression, IFN-α has been extensively utilized in the clinic as an adjuvant treatment for melanoma patients at high risk of relapse after surgical resection. IFN-α exerts its antitumor effects via different mechanisms, including immunoregulation and shifting host immunity from a Th2 predominant response to a Th1 response^33^, resulting in improved disease-free survival. In a genetically engineered mouse melanoma model, targeted activation of IFN-α in combination with blockade of PD-1 was shown to prolong survival significantly^34^. As shown in Fig. 2, IFN-γ treatment predominantly induced nNOS expression in melanoma cells, which were inhibited by IFN-α. The distinct effects of IFN-α and IFN-γ on regulating nNOS-NO signaling may help to explain the distinct clinical responses of the two isotype IFNs in melanoma patients, which also provides new insight into the pathogenesis of IFN-γ-stimulated melanoma progression.

IFN-γ, produced mainly by natural killer (NK) cells and natural killer T (NKT) cells as part of the innate response, before antigen-specific immunity develops, is crucial for immune response^35^. Secreted IFN-γ mediates the function of antigen presenting cells (APCs), inhibits Th2 cell development, and promotes the differentiation of Th1 cells, which further increases IFN-γ secretion^36^. Studies of healthy individuals have shown that with exposure to UV radiation, especially in the event of a sunburn, macrophages are recruited to the area and secrete IFN-γ, leading to significantly increased serum IFN-γ levels which remain elevated for several weeks^37^. In a UVB-HGF/SF transgenic mouse model, blocking IFN-γ effectively abolished macrophage-enhanced melanoma growth and survival^15^. Even though IFN-γ-mediated NO production by macrophages plays a pivotal role in the protective immunity against microbial pathogens^38^, earlier murine studies showed that T cell-derived IFN-γ activates the production of NO, which suppresses T cell proliferation by initiating a cycle of macrophage activation^39^. Consistently, other studies showed that the upregulation of NO was associated with immune suppression^40^, which may, at least partially, contribute to UV-induced local immunosuppression^41^ and the stimulation effects of NO on cell proliferation and invasion potential observed in cancers^7,24^. In recent years, accumulating evidence indicates that IFN-γ may alter the microenvironment of cancer cells, which allows them to escape from an immune response. This effect may be explained by the suppression of T lymphocyte proliferation by the nNOS-NO axis^39,42,43^.

Moreover, our RPPA results showed that IFN-γ treatment induced the expression of genes associated with poor prognosis and disease progression in melanoma patients, such as PD-L1, c-Myc, and HIF1α^21,44^. In recent years, more and more studies demonstrate the critical role of IFN-γ in melanoma immunity via upregulating PD-L1 expression, fostering an immune-suppressive microenvironment^18,45^. Ribas’ group at UCLA found that the regulation of PD-L1 expression is through the JAK1/2-STAT1/3-IRF1 axis^19^. As a primary inducer of PD-L1 expression^18,31^, IFN-γ was detected at the interface of PD-L1^+^ tumors and tumor infiltrating lymphocytes (TIL), suggesting that TILs trigger their own inhibition by secreting cytokines such as IFN-γ that drive tumor PD-L1 expression^46^. Though IFN-γ was found to be a cytotoxic T lymphocyte (CTL) chemoattractant that increases CTL cytotoxic function and motility^47^, melanoma cells seem to have acquired the ability to hijack the IFN-γ signaling pathway to upregulate PD-L1, thus escaping the immune response. Our study demonstrated that nNOS inhibitors effectively inhibited the induction of PD-L1 by IFN-γ in melanoma cells and reduced PD-L1 expression in xenograft tumors. The important role of nNOS-NO signaling in IFN-γ-stimulated melanoma progression and PD-L1-mediated immunosuppression provides a unique strategy for adjuvant treatment of melanoma by targeting the IFN-γ-nNOS-NO-PD-L1 signaling axis (Fig. 6).

Nuclear transcription factors STAT1 and STAT3 are well known downstream targets activated by IFN-γ^48^. In general, STAT1 is considered a tumor suppressor^48^, but there is growing evidence showing that overactivated STAT1 can also act as a tumor promotor^49^. Knockdown STAT1 in melanoma was shown to slow the migration and invasion potential both in vitro and in vivo^50^. STAT3 has also been implicated in the regulation of many genes that contribute to the signaling pathways in melanoma survival and proliferation^48,51^. An earlier study showed that STAT3 activated by IFN-γ serves as an important transcription factor of PD-L1 expression^52^. Recent mechanistic studies demonstrated that in human melanoma cells, PD-L1 expression was primarily regulated by the IFN-γ-activated JAK1/2-STAT1/2/3-IRF1 axis^19^. Such adaptive induction of PD-L1 in response to IFN-γ represents a novel mechanism by which cancer cells attempt to protect themselves from immune cell-mediated killing^45^. A loss-of-function mutation of IFN-associated JAK signaling results in acquired resistance of PD-L1 blockade with a lack of response to IFN-γ-induced PD-L1^19,53^. Thus, a detailed understanding of signaling pathways regulating the induction of PD-L1 by IFN-γ may help to improve anti-cancer immunity. Our study demonstrated that both the expression levels and activation of STAT1 and STAT3 in melanoma were increased after IFN-γ treatment, which were abolished by co-treatment with nNOS inhibitors. The data suggests that nNOS-NO signaling may play an important role in IFN-γ-activated STAT1/3 signaling and inhibition of nNOS using specific inhibitors may further disrupt the IFN-γ-activated STAT-PD-L1 trajectories.

Developing a small molecule to rescue immune response in cancer patients has attracted increasing attention among researchers due to their unique advantages. Small molecules, unlike biologics, are more stable and may be administered orally, and because of their smaller size, the biodistribution and in vivo delivery may be superior compared to monoclonal antibodies. There are also lower costs associated with the production, preparation, and drug delivery of small molecules without the severe immune-related adverse events observed in patients receiving treatment with biologics^54^. In recent years, researchers have developed many highly potent and selective indoleamine 2,3-dioxygenase (IDO1) inhibitors, such as epacadostat, that restores IL-2 production, resulting in direct reactivation of T cells^55^. Although, in a recent Phase III clinical trial, when combined with anti-PD-1 pembrolizumab, epacadostat failed to demonstrate a significant improvement of survival in melanoma patients, developing small molecules to regulate immune response remains an attractive approach for immunotherapy^56^. Our study demonstrated, for the first time, that nNOS inhibitors effectively inhibit IFN-γ-inducible PD-L1 both in vitro and in vivo. These observations provide support for the use of nNOS-selective inhibitors to rescue PD-L1-mediated immunosuppression in melanoma patients. Our approach of targeting nNOS-mediated NO signaling may be complementary to and potentially synergistic with the use of antibody-based immunotherapy as well as the conventional chemotherapy.

Taken together, our study demonstrates that targeting nNOS-NO signaling using nNOS-selective inhibitors may be an effective strategy for melanoma treatment, given its novel mechanism of action, not only inhibiting nNOS-stimulated melanoma progression by reducing the production of NO, but also by inhibiting IFN-γ-activated STAT1/3 and PD-L1.

## Materials and methods

### Cell lines, chemicals, and reagents

The human melanoma cell lines A375, wm115, and Sk-Mel-28 were obtained from American Type Culture Collection (Manassas, VA), and wm3211 was obtained from Rockland Immunochemicals (Limerick, PA). Cell lines were cultured in Dulbecco’s Modified Eagle’s Medium (DMEM; #11995073; Gibco, Waltham, MA) (A375) or Eagle’s Minimum Essential Medium (EMEM) (wm115, Sk-Mel-28) with 10% fetal bovine serum (FBS; #26140079; Gibco, Waltham, MA), or Tumor Specialized Media with 2% FBS (wm3211). Human immortal melanocyte cell lines (Hermes 1, 3a and 4a) were generously provided by Professor Dorothy C. Bennett (University of London, UK). The culture media and conditions followed the directions as provided by The Wellcome Trust Functional Genomics Cell Bank^27^. Human IFN-γ was purchased from GoldBio (1160-06-100; St. Louis, MO) and IFN-α was obtained from PBL Assay Science (11100-1; Piscataway, NJ).

### Antibodies

Mouse monoclonal anti-human NOS1 (nNOS) (MAB2416-SP, Novus Biologicals, Centennial, CO), STAT3, p-STAT3, Lamin A/C (sc-8019; sc-8059; sc-398927; Santa Cruz Biotechnology, Dallas, TX), rabbit monoclonal STAT1 (9175S;Cell Signaling Technology, Danvers, MA), and mouse monoclonal anti-human β-Actin (8H10D10; Cell Signaling Technology, Danvers, MA) antibodies were used as primary antibodies; horseradish peroxidase-labeled anti-mouse or anti-rabbit (Cell Signaling Technology, Danvers, MA) were used as the secondary antibodies. Rabbit monoclonal PD-L1 conjugated with Alexa Fluor 488 (25048, Cell Signaling Technology, Danvers, MA) was used for extracellular expression analysis via flow cytometry and immunofluorescence.

### Matrigel invasion assay

Matrigel invasion assay was conducted using a cell culture insert precoated with extracellular matrix proteins (354480, Corning). Briefly, a cell suspension (10,000 cells in 100 μL) in DMEM with 1% FBS was added to the upper chamber. Next, 500 μL of DMEM containing IFN-γ was added to the lower chamber of the Transwell. The cells were allowed to migrate for 16 h at 37 °C. After incubation, the non‐migrated cells were removed from the upper surface via scraping. The filter was then fixed and stained with crystal violet. The average of all cells that had migrated from the upper to the lower side of the filter was quantified using a light microscope. This experiment was repeated three times. The number of migrated cells was determined by the average cell number of 10 fields of view.

### Detection of intracellular nitric oxide levels

Cultured cells were treated with IFN-α or IFN-γ with or without nNOS inhibitors at various timepoints in serum free medium. The medium was then replaced with Hank’s Balanced Salt Solution (HBSS), followed by the addition of 4-Amino-5-methylamino-2’,7’-difluorofluorescein (DAF-FM) (1 μM). Fluorescence levels were detected using flow cytometry or a fluorescence microplate reader with excitation and emission wavelengths of 485 and 538 nm, respectively.

### Protein isolation and western blotting

Whole cell lysates were collected by incubating cell suspensions in lysis buffer (9803S; Cell Signaling, Danvers, MA) with 1% protease inhibitor cocktail (PIC) for 15 min then lysed via shear force. Protein samples were isolated using centrifugation at 14,000 g for 10 min at 4 °C.

Equal amounts of protein were resolved on 8% SDS–polyacrylamide gels, then transferred to PVDF membranes. Membranes were cut into strips, blocked using 10% non-fat milk or 5% bovine serum albumin, then incubated with primary antibodies for 1 h at room temperature or overnight at 4 °C, followed by secondary antibodies for 1 h at room temperature as recommended by the manufacturers. Labeled bands were detected using SuperSignal horseradish peroxidase chemiluminescence reagents (1859674; 1859675; Thermo Fisher Scientific, Waltham, MA), and images were captured and analyzed using the Bio-Rad ChemiDoc XRS^+^ System.

### Reverse phase protein array

Cells were treated with 250 units/mL of IFN-α or IFN-γ for 48 h. Lysis buffer (1% Triton X-100, 50 mM HEPES, pH 7.4, 150 mM NaCl, 1.5 mM MgCl_2_, 1 mM EGTA, 100 mM NaF, 10 mM Na pyrophosphate, 1 mM Na_3_VO_4_, 10% glycerol and 1% PIC) was added to the plates, followed by incubation on ice for 20 min with occasional shaking. Samples were then collected via scraping and spun down at 14,000 g for 10 min at 4 °C. The protein concentration was then quantified and adjusted to 1.5 µg/µL with lysis buffer. Cell lysates were then mixed with 4 × SDS sample buffer (40% glycerol, 8% SDS, 0.25 M Tris–HCl, pH 6.8 with 10% BME). Miniscule amounts of serially diluted protein samples were then dotted on a nitrocellulose-coated slide and probed with validated primary antibodies and a biotin-conjugated secondary antibody. The signals were amplified using Dako-Cytomation-Catalyzed system (Dako) and visualized by diaminobenzidine colorimetric reaction. Dilution curves were fitted with Supercurve Fitting and the protein expressions normalized for protein loading. The heatmaps included in supplemental data were generated in Cluster 3.0 (http://bonsai.hgc.jp/~mdehoon/software/cluster/software.htm) as a hierarchical cluster using Pearson Correlation and a center metric.

### In vitro cytotoxicity of nNOS inhibitors detected by MTT colorimetric assay

Cell viability was determined by MTT as described previously^57^. Three human melanoma cell lines (A375, Sk-Mel-28, and wm-3211) and three human immortal melanocyte cell lines (Hermes 1, 3a and 4a) were utilized to detect the cytotoxicity of tested nNOS inhibitors. After 72 h of treatments, MTT solution was added to each well to a final concentration of 0.5 mg/mL. Formed crystals were solubilized and the absorbance was measured at 595 nm. *GraphPad Prism 7* was used to determine the IC_50_ of each compound by plotting percent cell viability against the log drug concentrations and fitted using a nonlinear fit of the normalized data.

### Expression levels of PD-L1 detected by flow cytometry and immunofluorescence

A375 cells were incubated with 250 units/mL of IFN-α or IFN-γ in the presence or absence of 3 μM of nNOS inhibitors for 48 h. Cells were collected and fixed using 4% formaldehyde in PBS for 10 min at 37 °C. The cells were then washed with incubation buffer (0.5% BSA in PBS), followed by incubation with PD-L1 antibody in the dark for 2 h at room temperature. Mean fluorescence intensities were measured via flow cytometry and recorded for analysis.

After treatment, cells were fixed with 4% formaldehyde/PBS for 15 min at room temperature, followed by incubation with ice-cold 100% methanol for 10 min at -20 °C. Samples were then incubated in blocking buffer (PBS, 5% horse serum, 0.3% Triton X-100) for 1 h, then in PD-L1 antibody in dilution buffer (PBS, 1% BSA, 0.3% Triton X-100) overnight at 4 °C. Stained specimens were rinsed with PBS then cured in the dark with DAPI fluorescence staining reagent for 1 h. Slides were visualized and recorded using the BZ-X700 microscope (Keyence, Itasca, IL).

### In vivo xenograft melanoma mouse model

The study was carried out in compliance with the ARRIVE guidelines. All of the animal procedures were approved by the Institutional Animal Care and Use Committee (IACUC) at Chapman University and conducted in compliance with the policies of Chapman University, and federal, state, and local animal welfare authorities. Nude mice (*Nu/Nu*) were purchased from Charles River (Wilmington, MA) and were housed and maintained in the Chapman University vivarium under pathogen-free conditions. A375 cells were suspended in cold Matrigel (354248; Corning, Corning, NY) and injected subcutaneously into the flank of the mouse (1 × 10^6^ cells per mouse) to establish tumors. The mice were treated with intraperitoneal injections of normal saline or IFN-γ (1000 units/mouse) with or without MAC-3-190 (5 mg/kg/day) or HH044 (10 mg/kg/day) for 21 days. The growth of the tumors was monitored three times a week and measured using digital Vernier calipers. Tumor volume (mm^3^) was calculated as [Length × (Width^2^)]/2. The mice were sacrificed after 21 days, and tumors and lungs were removed and weighed. Half of the tumor was fixed in a 10% formalin solution and the other half was immediately processed for flow cytometry. The fixed samples were then further embedded in paraffin wax for sectioning and stained with specific PD-L1 (790–4905; Ventana, Oro Valley, AZ) and CD8 (108M-98; Cell Marque, Rocklin, CA) antibodies using the Ventana Benchmark Ultra. The percentage of PD-L1 stained positive cells were counted using ImageJ (https://imagej.nih.gov/ij/index.html). Tumor samples for flow cytometry were dissociated into a single cell suspension using the gentleMACS Dissociator from Miltenyi Biotec (130-095-929; Auburn, CA) following the standard protocol for soft tumors. Single cell suspensions were then fixed and stained with PD-L1 antibodies as described above.

### Statistical analyses

All the experiments were repeated at least twice and performed in at least two different human melanoma cell lines. Data shown are means ± SD from a representative of at least two independent experiments. Statistical analysis was performed by using the student *t*-test and a *p* value of less than 0.05 was considered statistically significant.

## Supplementary Information

Supplementary Information 1.

Supplementary Information 2.

Supplementary Information 3.

Supplementary Information 4.
