# Supplementary figures and images for: Inhibition of interferon-gamma-stimulated melanoma progression by targeting neuronal nitric oxide synthase (nNOS)
Source: Sci Rep. 2022 Feb 1;12:1701. doi: 10.1038/s41598-022-05394-6 (PMC8807785; doi:10.1038/s41598-022-05394-6)

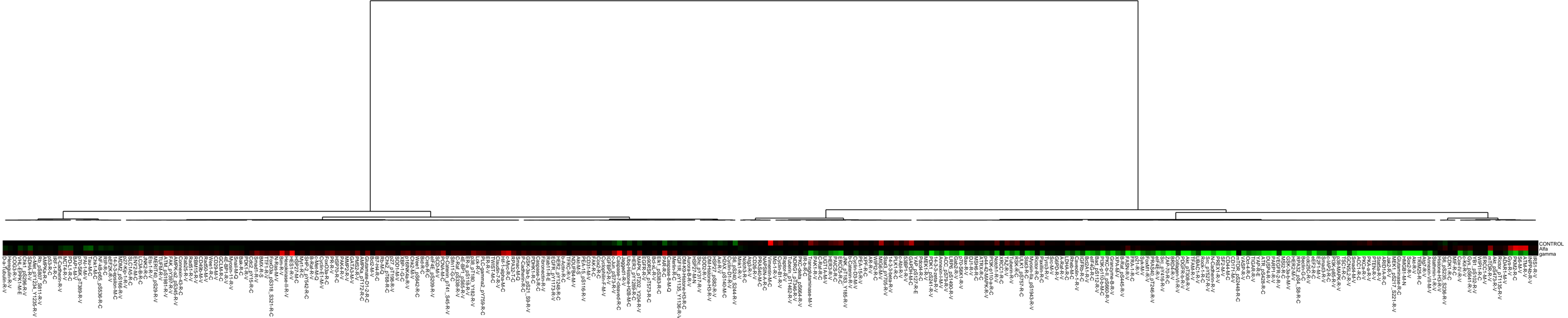

CONTROL  
Alfa  
gamma

Supplement: Supplementary file 1 — Supplementary Information 1. [file 41598_2022_5394_MOESM1_ESM.pdf]

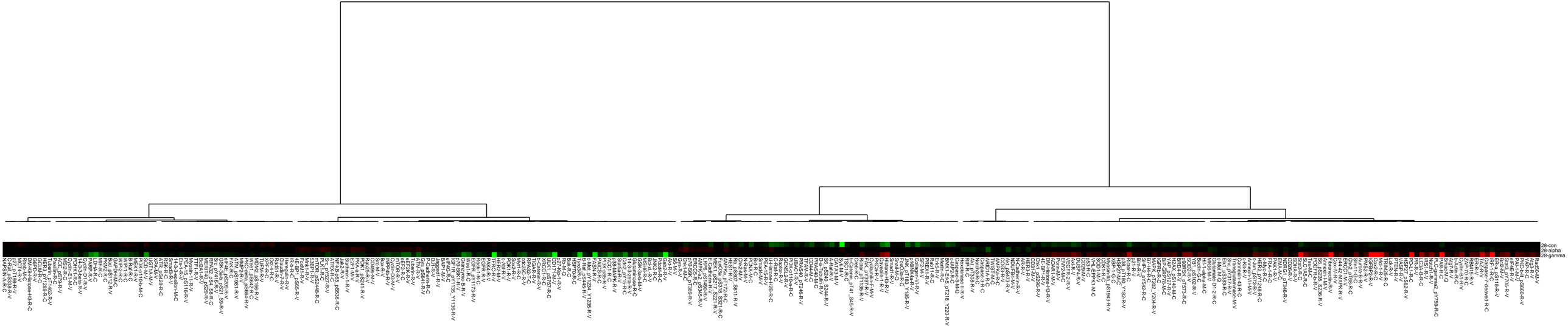

Supplement: Supplementary file 2 — Supplementary Information 2. [file 41598_2022_5394_MOESM2_ESM.pdf]

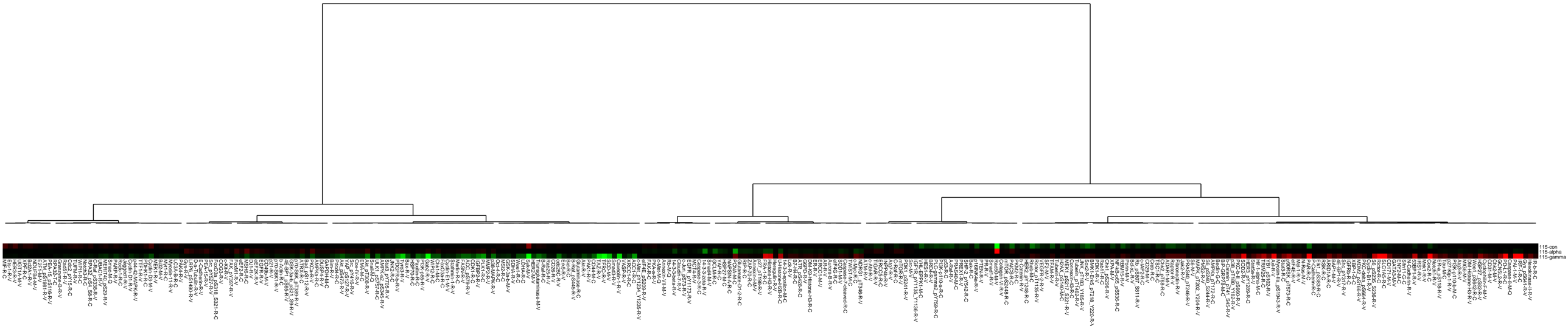

Supplement: Supplementary file 4 — Supplementary Information 4. [file 41598_2022_5394_MOESM4_ESM.pdf]
