# Supplementary material for: Inhibition of interferon-gamma-stimulated melanoma progression by targeting neuronal nitric oxide synthase (nNOS)
Source: Sci Rep. 2022 Feb 1;12:1701. doi: 10.1038/s41598-022-05394-6 (PMC8807785; doi:10.1038/s41598-022-05394-6)
Supplement: Supplementary file 3 — Supplementary Information 3. [file 41598_2022_5394_MOESM3_ESM.pptx]

## Slide 1
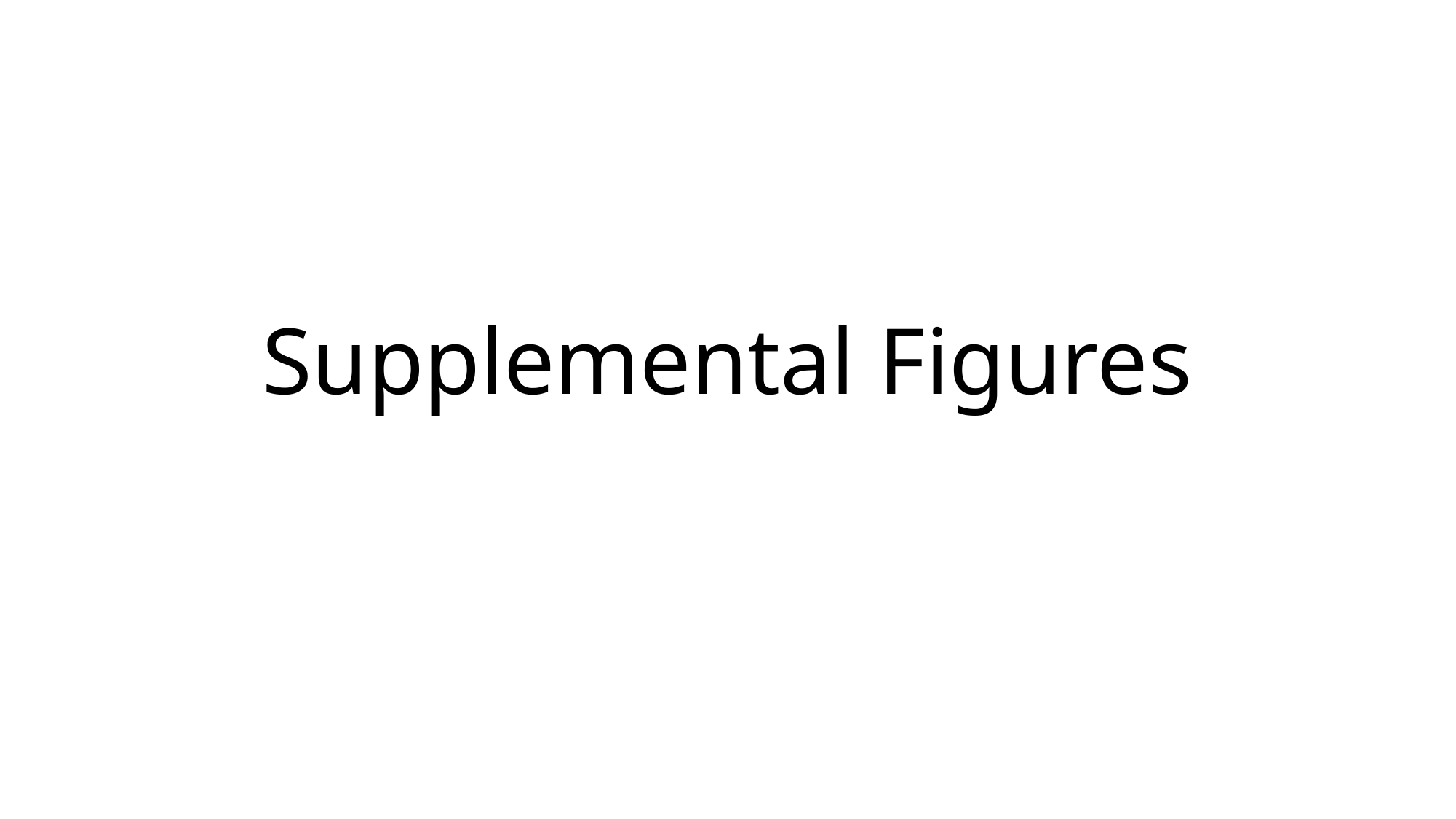

# Supplemental Figures

## Slide 2
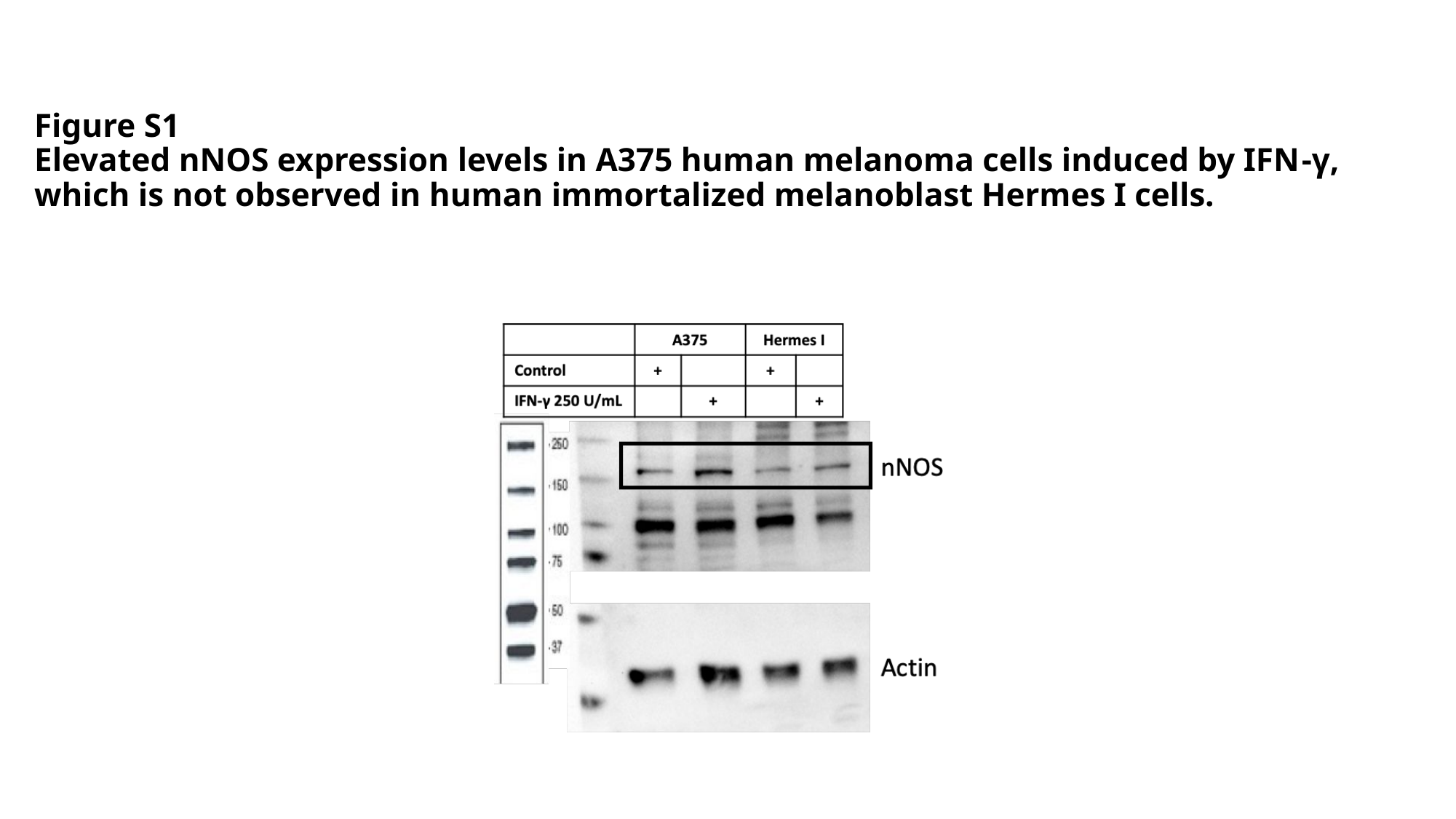

# Figure S1 Elevated nNOS expression levels in A375 human melanoma cells induced by IFN-γ, which is not observed in human immortalized melanoblast Hermes I cells.

## Slide 3
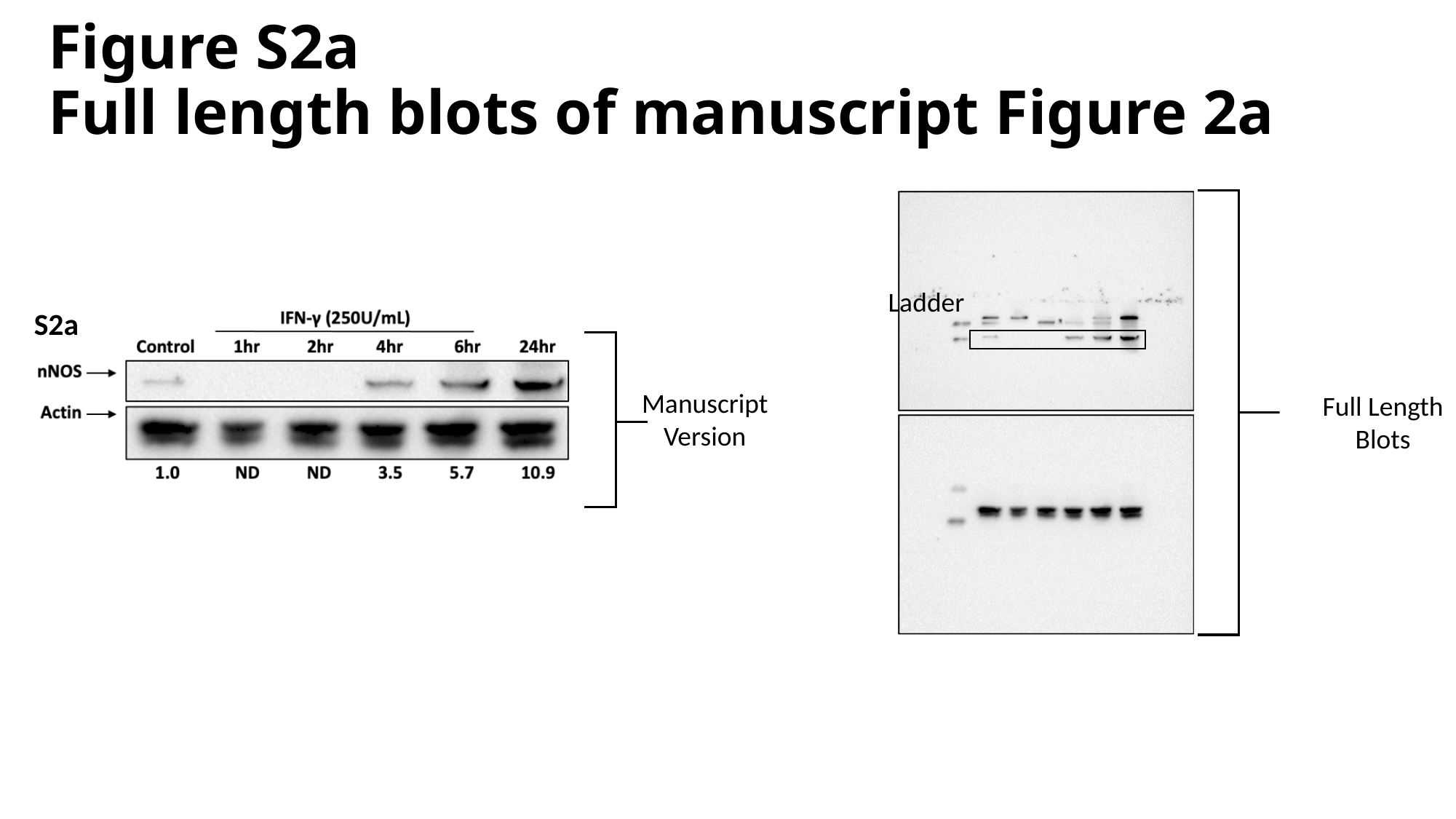

# Figure S2a Full length blots of manuscript Figure 2a
Ladder
S2a
Manuscript Version
Full Length
Blots

## Slide 4
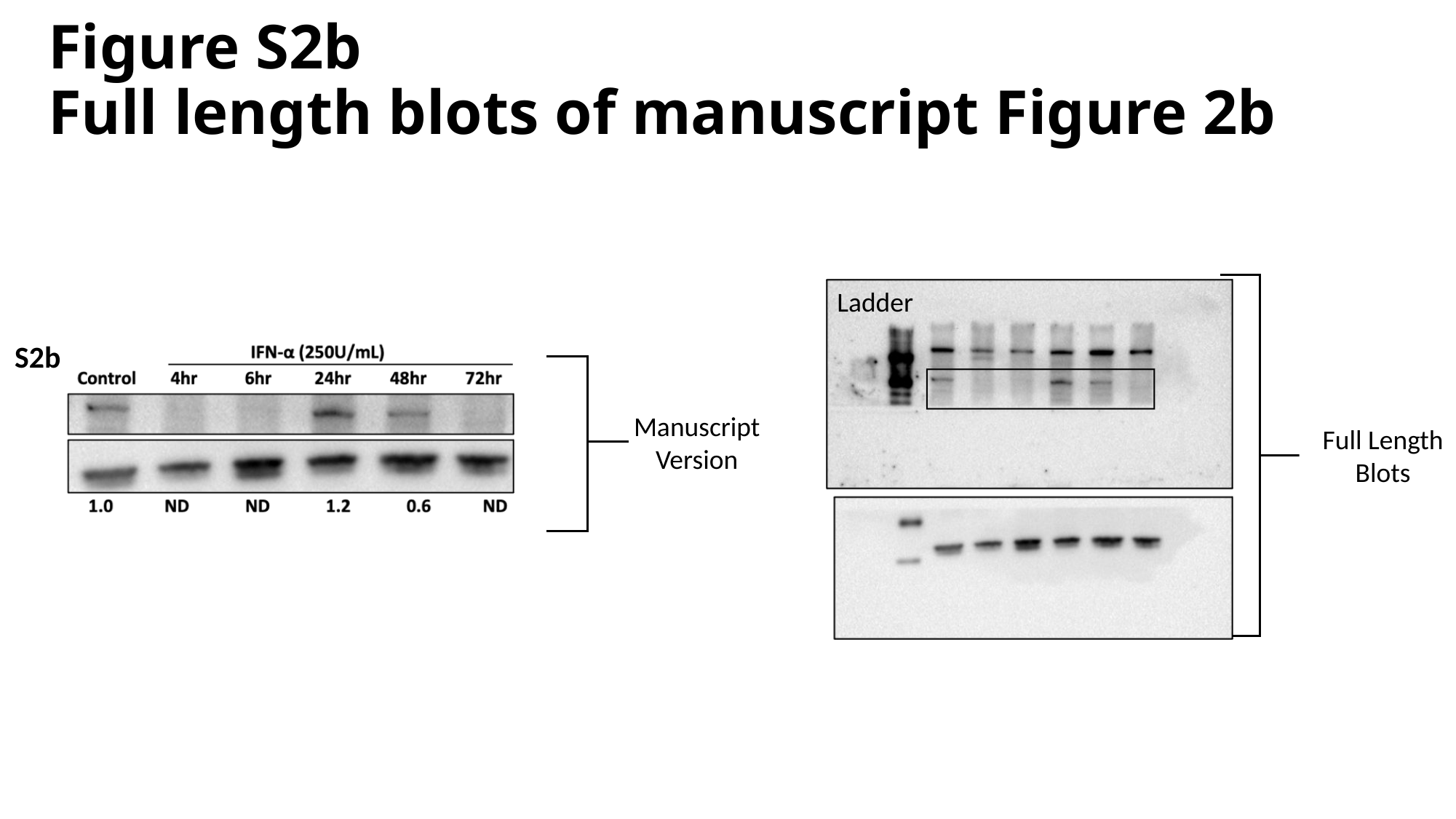

# Figure S2b Full length blots of manuscript Figure 2b
Ladder
S2b
Manuscript Version
Full Length
Blots

## Slide 5
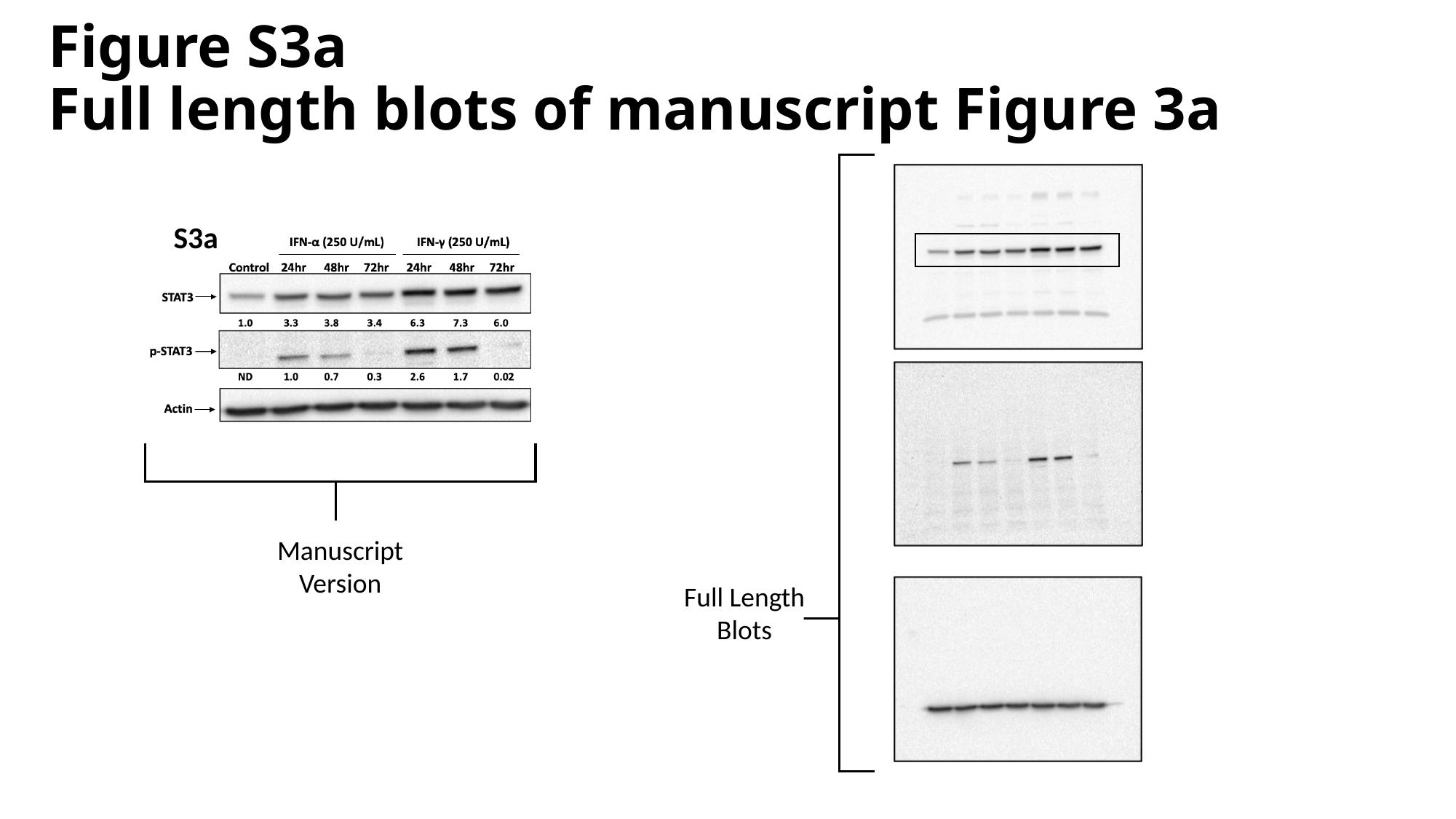

# Figure S3a Full length blots of manuscript Figure 3a
S3a
Manuscript Version
Full Length
Blots

## Slide 6
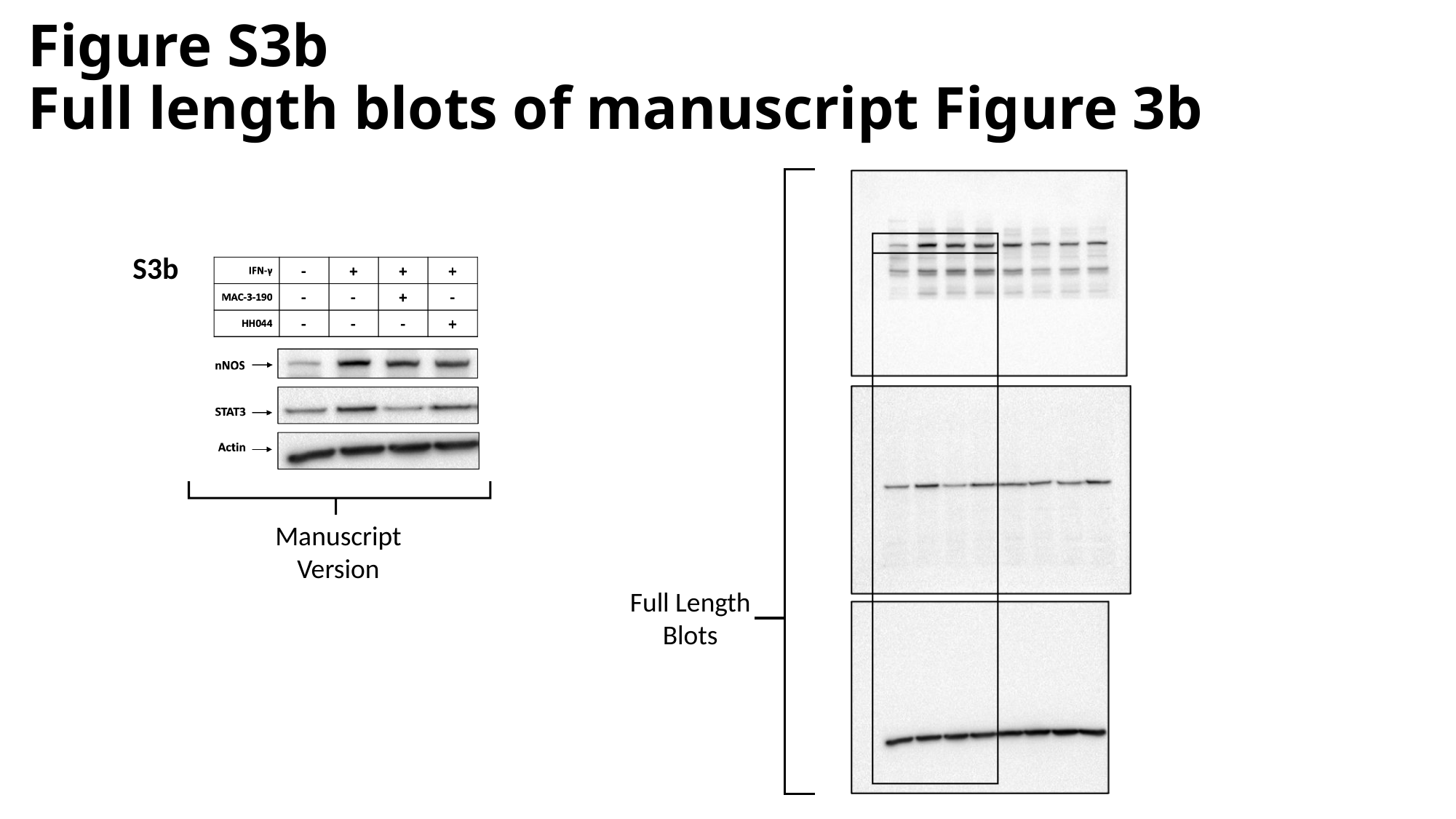

# Figure S3b Full length blots of manuscript Figure 3b
S3b
Manuscript Version
Full Length
Blots

## Slide 7
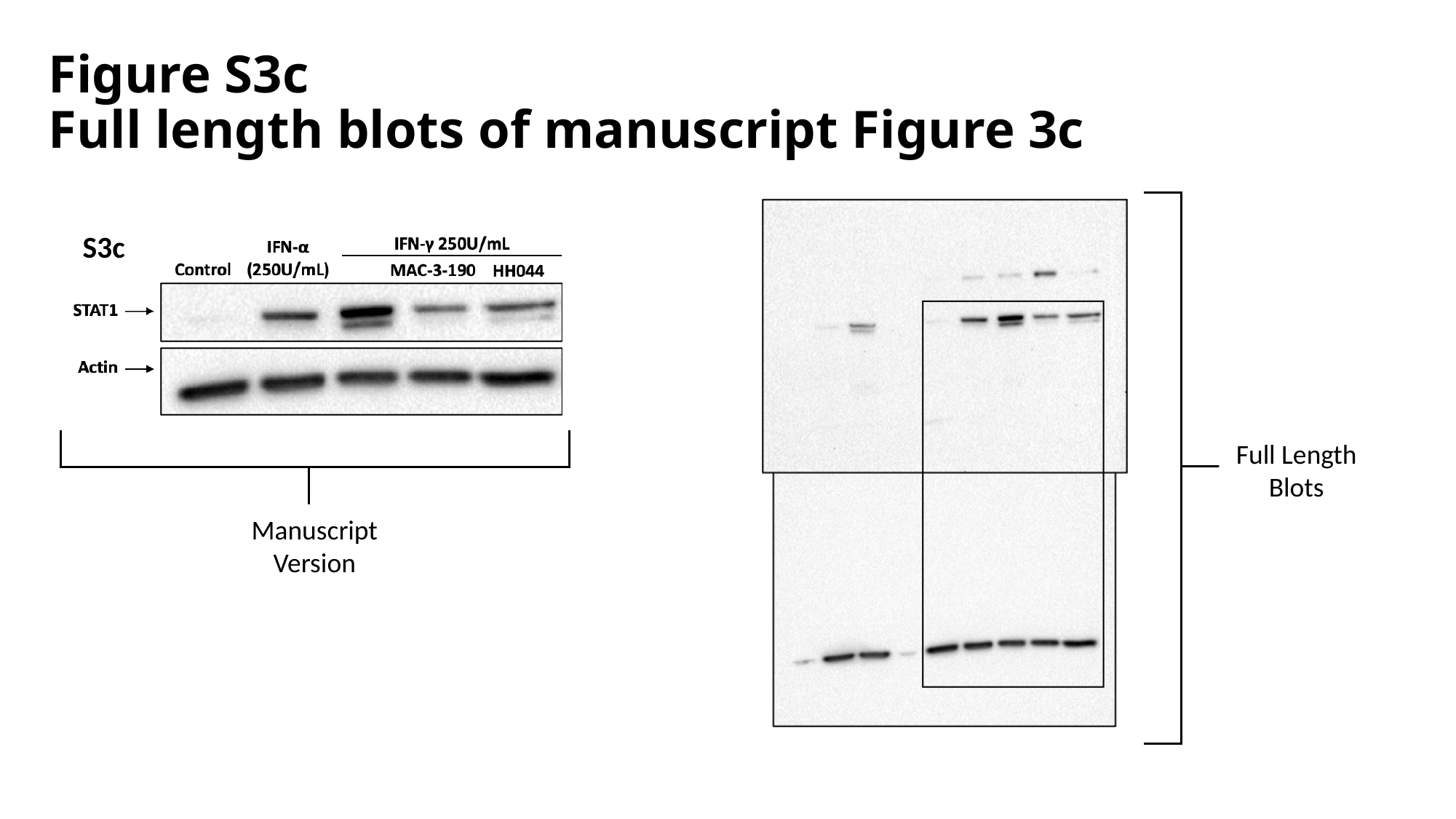

# Figure S3c Full length blots of manuscript Figure 3c
S3c
Full Length
Blots
Manuscript Version

## Slide 8
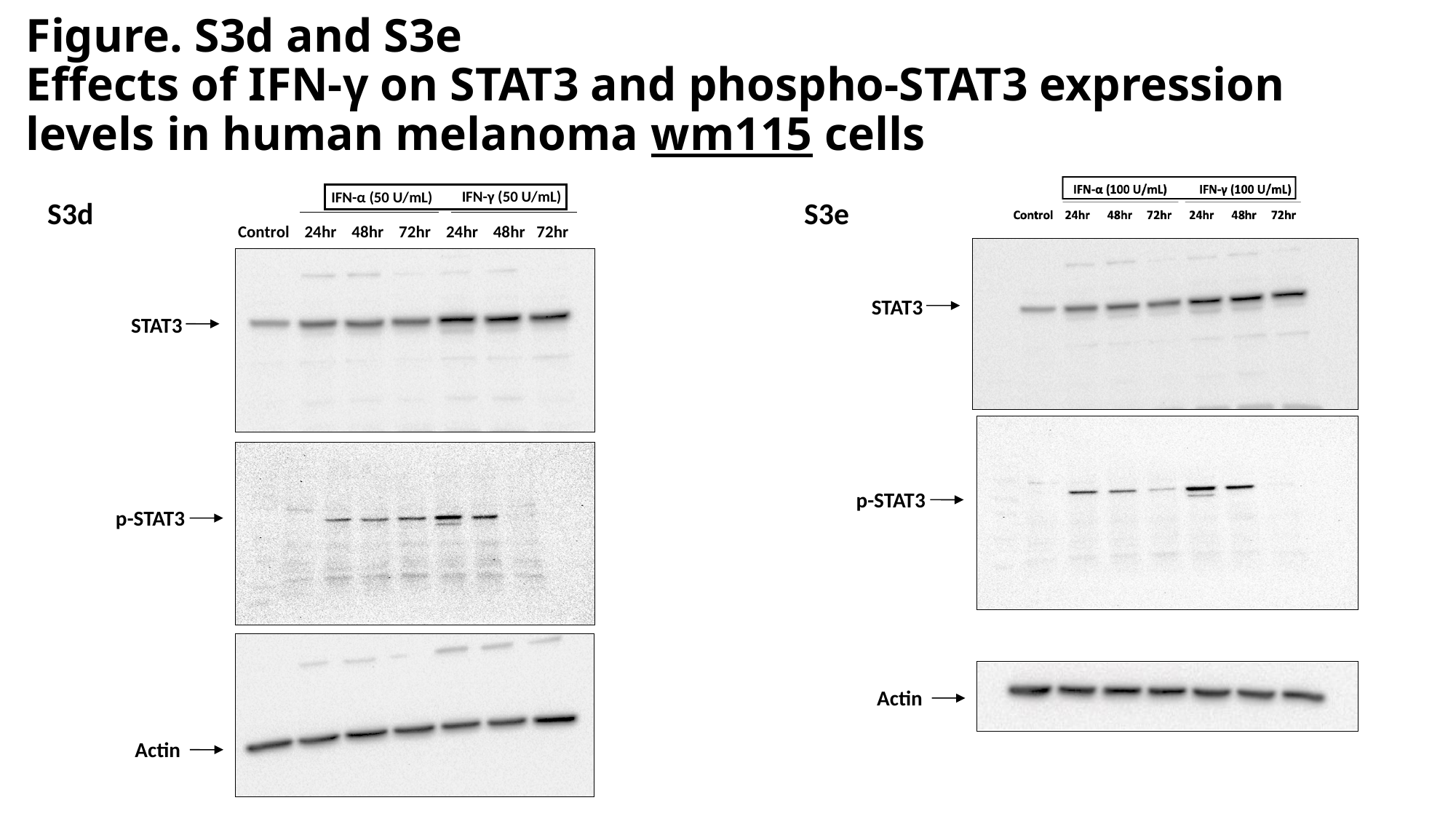

Figure. S3d and S3e
Effects of IFN-γ on STAT3 and phospho-STAT3 expression levels in human melanoma wm115 cells
IFN-γ (50 U/mL)
IFN-α (50 U/mL)
S3d
S3e
Control 24hr 48hr 72hr 24hr 48hr 72hr
STAT3
STAT3
p-STAT3
p-STAT3
Actin
Actin

## Slide 9
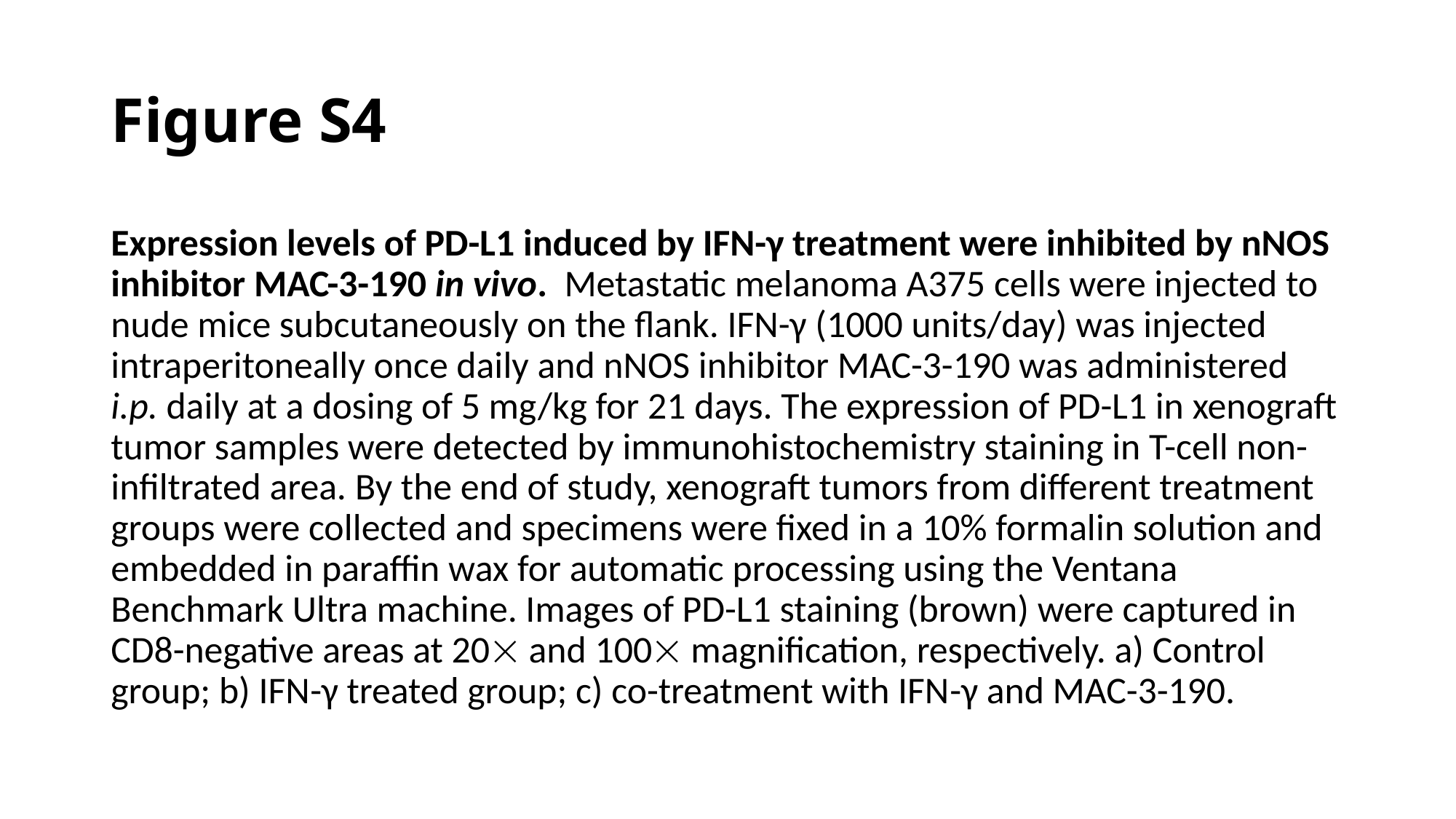

# Figure S4
Expression levels of PD-L1 induced by IFN-γ treatment were inhibited by nNOS inhibitor MAC-3-190 in vivo. Metastatic melanoma A375 cells were injected to nude mice subcutaneously on the flank. IFN-γ (1000 units/day) was injected intraperitoneally once daily and nNOS inhibitor MAC-3-190 was administered i.p. daily at a dosing of 5 mg/kg for 21 days. The expression of PD-L1 in xenograft tumor samples were detected by immunohistochemistry staining in T-cell non-infiltrated area. By the end of study, xenograft tumors from different treatment groups were collected and specimens were fixed in a 10% formalin solution and embedded in paraffin wax for automatic processing using the Ventana Benchmark Ultra machine. Images of PD-L1 staining (brown) were captured in CD8-negative areas at 20× and 100× magnification, respectively. a) Control group; b) IFN-γ treated group; c) co-treatment with IFN-γ and MAC-3-190.

## Slide 10
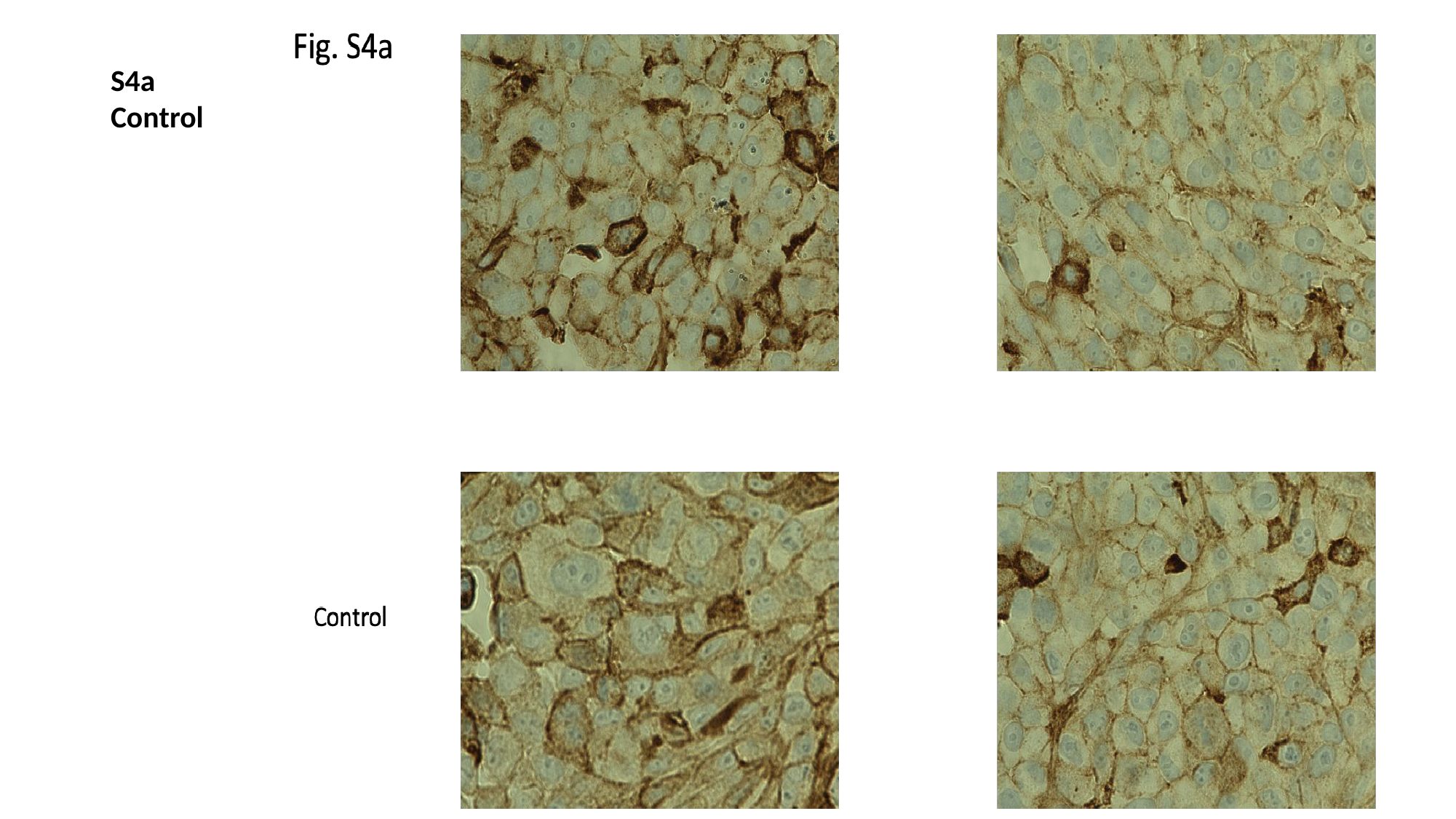

S4a
Control

## Slide 11
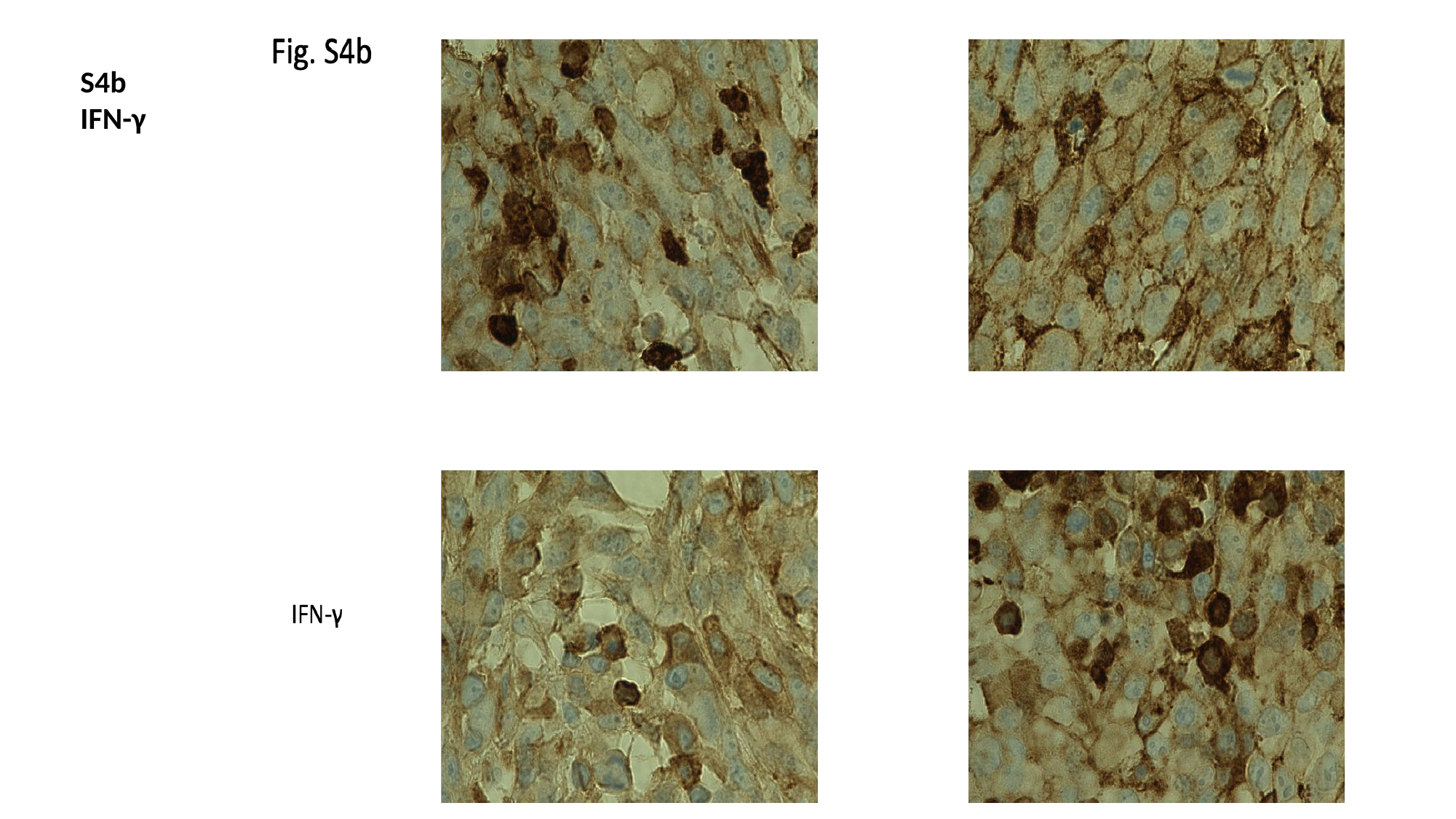

S4b
IFN-γ

## Slide 12
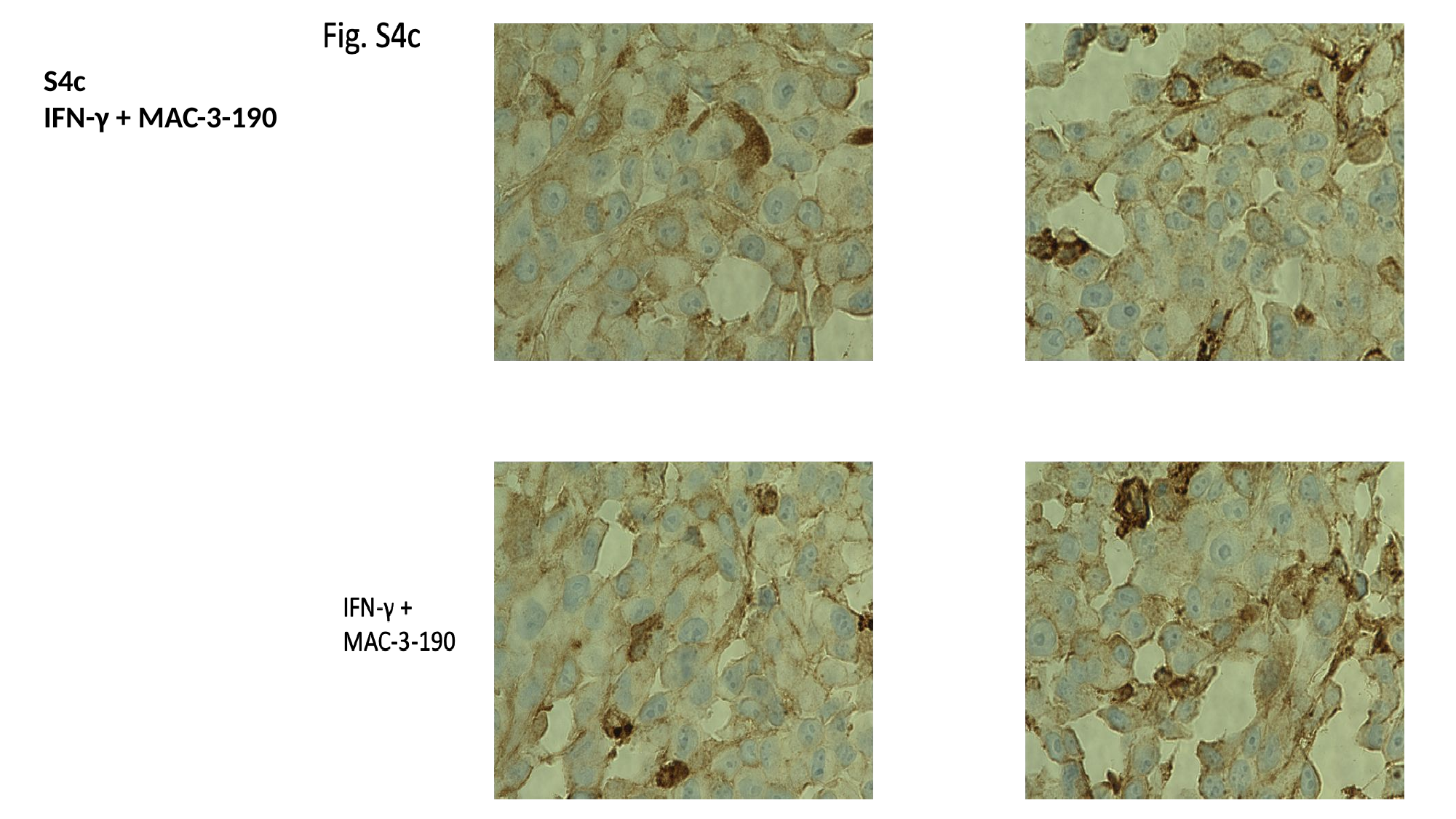

S4c
IFN-γ + MAC-3-190

## Slide 13
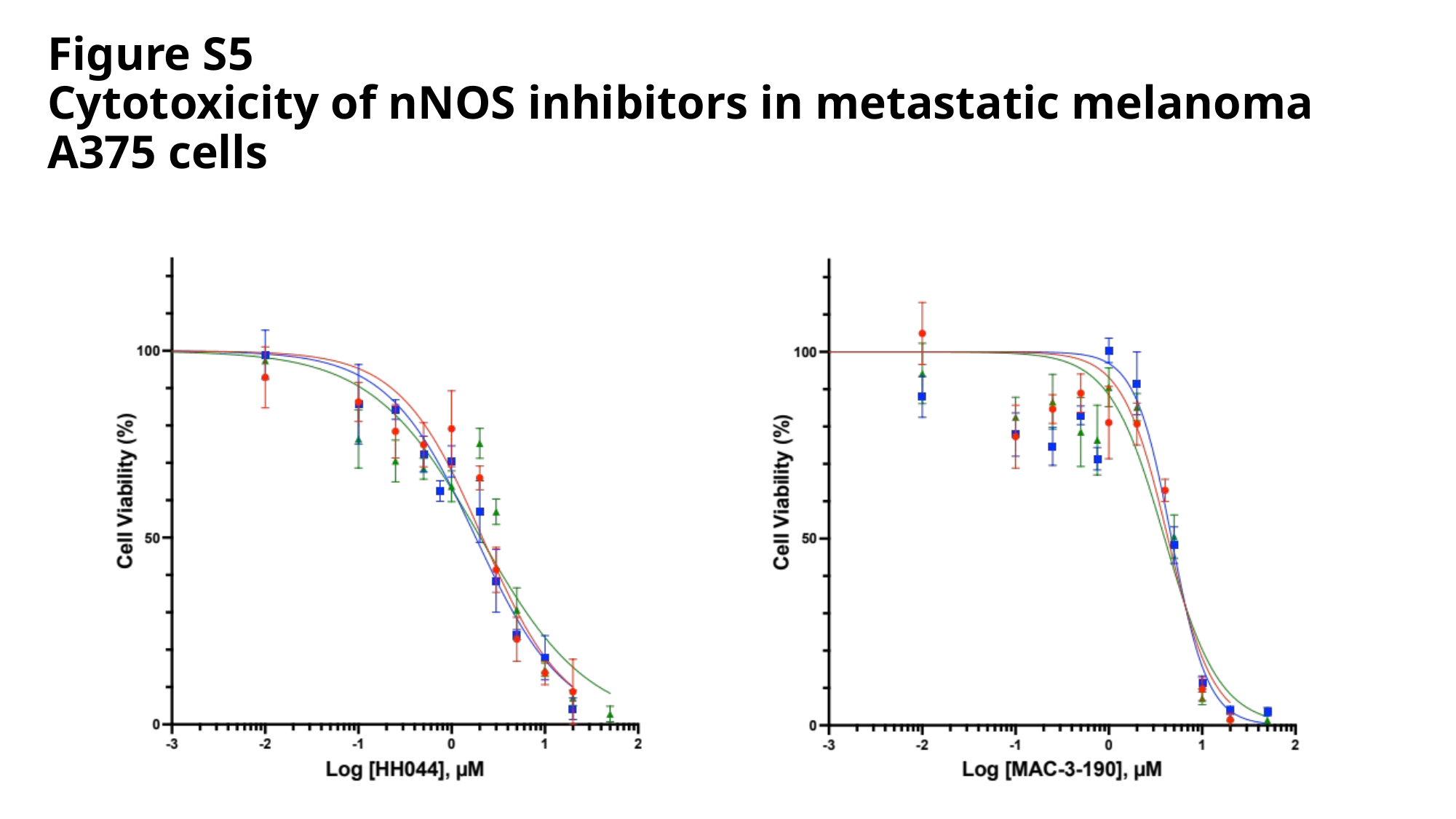

# Figure S5 Cytotoxicity of nNOS inhibitors in metastatic melanoma A375 cells
